# Effects of soy isoflavones on menopausal symptoms in perimenopausal women: a systematic review and meta-analysis

**DOI:** 10.7717/peerj.19715

**Published:** 2025-07-23

**Authors:** Haodi Luan, Qianqian Liu, Yahui Guo, Hua Fan, Sileng A., Jing Lin

**Affiliations:** 1 Clinical Nutrition Department, Critical Care Medicine Department, The Affiliated Hospital of Inner Mongolia Medical University, Hohhot, China; 2 Department of Gastroenterology, The Affiliated Hospital of Inner Mongolia Medical University, Hohhot, China; 3 Medical Simulation Center, Inner Mongolia Medical University, Hohhot, China

**Keywords:** Climacteric, Soy isoflavones, Menopause, Phytoestrogens, Hot flush, Meta-analysis

## Abstract

Soy isoflavones are phytoestrogens found mainly in soy and its derivatives. Given their estrogen-like and antioxidant-inhibiting inflammatory effects, they have been hypothesized to be effective in treating menopausal symptoms. We conducted a systematic review in accordance with the PRISMA guidelines. In October 2024, we screened 2,099 articles, of which 12 were eligible for meta-analysis, and found that soy isoflavones were effective for treating menopausal symptoms (seven studies, 533 participants, Hedges’ g = −0.25, 95% CI [−0.42 to −0.08], *p* = 0.00). Soy isoflavones showed significant effects on headache (three studies, 340 participants, Hedges’ g = −0.38, 95% CI [−0.60 to −0.17], *p* = 0.00), psychosocial symptoms (five studies, 416 participants, Hedges’ g = −0.29, 95% CI [−0.48 to −0.10], *p* = 0.00), palpitation (three studies, 356 participants, Hedges’ g = −0.42, 95% CI [−0.63 to −0.22], *p* = 0.00), and depression (four studies, 748 participants, Hedges’ g = −0.72, 95% CI [−1.17 to −0.28], *p* = 0.00), but no significant treatment effect on paresthesia symptoms, fatigue symptoms, physical symptoms, hot flushes, excessive sweating, insomnia, and vasomotor symptoms was observed. However, our results should be interpreted with caution owing to the small sample size. More trials should be conducted in the future to validate our findings.

## Introduction

Menopause is a natural biological process, marking the cessation of a woman’s reproductive ability. It is characterized by various physiological and psychological changes. A very common and distressing symptom of this transitional period is the occurrence of vasomotor symptoms such as hot flashes and night sweats (Avis et al., 2001). The reduction in estrogen levels during menopause can lead to various symptoms that have a substantial impact on a woman’s overall quality of life (Groeneveld et al., 1993). At present, hormone replacement therapy (HRT) is frequently used to alleviate menopausal symptoms such as hot flashes, night sweats, and mood fluctuations (Pop et al., 2023). However, despite its effectiveness, HRT is associated with several drawbacks, which include increased risk of cardiovascular disease (Cagnacci & Venier, 2019), breast cancer (Stoer et al., 2024), and thromboembolic events (Gomes & Deitcher, 2004). Many women show concerns regarding the long-term effects of HRT, resulting in a preference toward alternative therapies. Consequently, there is growing interest in exploring non-hormonal treatment options, such as the use of plant-based isoflavones, particularly those derived from soy, which may provide symptom relief without the associated risks of traditional HRT. Overall, the transition toward safer, more sustainable treatment options reflects a significant shift in menopausal symptom management among perimenopausal women (Kang et al., 2022; Nahas et al., 2007; Sohn et al., 2021).

Soy isoflavones, a group of phytoestrogens predominantly found in soybean and its derivatives, have emerged as a potential alternative therapy for managing menopausal symptoms. These compounds, which primarily include genistein, daidzein, and glycitein, share structural similarities with human estrogen (17β-estradiol) and can bind to estrogen receptors, particularly ER-β. These compounds can therefore exert weak estrogenic effects (Poschner et al., 2017). When estrogen levels decline in menopausal women, soy isoflavones bind to estrogen receptor sites in the body. This binding induces a mild estrogenic effect, helping compensate for hormonal deficiencies and alleviate common menopausal symptoms such as hot flashes, night sweats, and mood swings (Khapre, Deshmukh & Jain, 2022). Conversely, during periods of high estrogen levels, soy isoflavones competitively block these receptors, reducing the effects of excess estrogen (Viscardi et al., 2025). This dual modulatory mechanism helps regulate hormonal balance and reduces discomfort caused by hormonal fluctuations during menopause. Although this mechanism suggests promising therapeutic potential, the clinical efficacy of soy isoflavones remains controversial. Some studies report substantial improvements in vasomotor symptoms and other menopausal disorders, whereas others show negligible or no benefits (Chen, Ko & Chen, 2019; Chen, Lin & Liu, 2015; Gencturk, Bilgic & Kaban, 2024; Mainini et al., 2024). These inconsistencies may be attributed to various factors, including differences in isoflavone formulations, dosages, individual metabolism (particularly the ability to produce equol from daidzein), and genetic variations in the study population. In addition, concerns have been raised about potential risks, such as interactions with endocrine function and breast cancer risk, although current evidence suggests safety at typical dietary intake levels (Chen, Ko & Chen, 2019; Chen, Lin & Liu, 2015; Gencturk, Bilgic & Kaban, 2024; Mainini et al., 2024). The conflicting results from numerous clinical trials and meta-analyses have sparked ongoing debates about the role of soy isoflavones in managing menopausal symptoms among the scientific community. Existing meta-analyses primarily investigate the association between soy isoflavones and menopausal symptoms as a whole, without addressing their effects on individual symptoms (such as insomnia, depression, hot flashes, and fatigue).

We aim to elucidate the role of soy isoflavones in managing menopausal symptoms, providing evidence-based guidance for women experiencing perimenopause, and fostering informed choices about dietary and supplementary options. By conducting a thorough systematic review and meta-analysis, we intend to deliver definitive insights into the effectiveness of soy isoflavones, by assessing the potential of these natural compounds to complement holistic strategies for women’s health during menopause.

## Methods

### Literature search strategy

We followed the PRISMA Statement guidelines to perform a systematic search (Moher et al., 2009). This study is also registered on the PROSPERO platform (CRD42024616691). The following databases were searched from inception to October 20, 2024: PubMed, Cochrane, Web of Science, and Embase. The following combinations of keywords were used during the search: soy, soya, flavones, flavonoids, genistein, daidzein, red clover, menopausal symptoms, and climacteric. Further, we hand-searched the reference lists of all included systematic reviews and meta-analyses to identify additional articles.

### Selection procedure

Studies were included if they met the following inclusion criteria: (1) a randomized controlled trial (RCT) design, including a parallel or crossover design; (2) inclusion of perimenopausal or postmenopausal women aged ≥35 years and experiencing menopausal symptoms; (3) inclusion of intervention group containing soy isoflavone (*e.g.*, soy extract, genistein, or isoflavone) and a control group with a placebo; (4) oral administration during the intervention; and (5) English language. The following studies were excluded: (1) observational studies, non-clinical trials, or animal studies; (2) studies where numerical outcome data were not provided; (3) studies that did not report on the specified outcome measures of interest.

### Data extraction

Two reviewers (Liu and Guo) independently and concurrently extracted data. Any uncertainties were resolved through consultation with a third reviewer (Luan). A standardized data extraction form was employed to gather information from the selected studies. We collected details regarding study characteristics, sample demographics, the type and duration of interventions, and the outcome measures. In addition, we obtained pre- and post-treatment means, standard deviations, participant counts, or pre-post mean differences and standard deviations for the purpose of meta-analysis. We also reached out to study authors *via* email to seek any missing data or clarifications, providing them with a tailored data table to facilitate the reporting of the requested information.

### Outcome measures

Outcome measures included menopause symptom-specific index scores (*e.g.*, Kupperman Index (Kupperman, Wetchler & Blatt, 1959), Greene Menopause Scale (Greene, 1998)), related menopausal symptoms, including hot flashes, insomnia, excessive sweating, headache, and fatigue, and quality of life scores (*e.g.*, MENQOL (Hilditch et al., 1996) and SF-36 (Larson, 1997)). An elevated score corresponded to a greater intensity and frequency of symptoms.

### Statistical analysis

We employed a random-effects model to compute the effect size. For continuous outcomes, we aggregated Hedges’ g for adjusting the effect size for small sample sizes. We calculated 95% confidence intervals for each effect size estimate. The assessment of heterogeneity among studies was conducted using the Cochrane Q and I^2^ statistics. The thresholds for interpreting heterogeneity aligned with those established by the Cochrane Collaboration (I^2^ 0–40%, possibly unimportant; 30–60%, potentially moderate heterogeneity; 50–90%, likely substantial heterogeneity; and 75–100%, significant heterogeneity) (Higgins & Cochrane Collaboration, 2019).

When multiple scales were used to measure the same symptom cluster, we selected the group that demonstrates superior data. When there were multiple treatment groups in a study (Costa et al., 2017; Fontvieille, Dionne & Riesco, 2017), we chose the group with soy isoflavone intervention plus exercise activities as the active treatment group, and the group with placebo alone plus exercise activities as the control group. One study (Jou et al., 2008) categorized participants into two groups based on whether their gut bacteria can break down and utilize soy isoflavones, producing the metabolite equol. In that study, we selected the data from the group that can metabolize and produce equol. For the assessment of menopausal symptoms, including hot flashes, hyperhidrosis, and insomnia, we used scoring systems rather than frequency counts. In these metrics, elevated scores corresponded to increased symptom severity.

We also examined publication bias *via* funnel plot analysis and used Egger’s regression test to quantify the potential asymmetry of the plot. To address the issue of missing data, we used multiple imputation techniques to estimate missing values, ensuring the robustness of our findings. Subgroup analyses were performed based on participant characteristics and baseline disease severity to evaluate differential treatment impacts across diverse groups. Sensitivity analyses were conducted to determine the robustness of the results by repeating the analyses after removing studies with a high risk of bias. Meta-regression analyses were also employed to identify potential sources of heterogeneity, such as study design, sample size, and duration of intervention. All statistical tests were two-sided and a *p*-value < 0.05 was considered statistically significant.

## Results

### Search results

**Figure 1:**
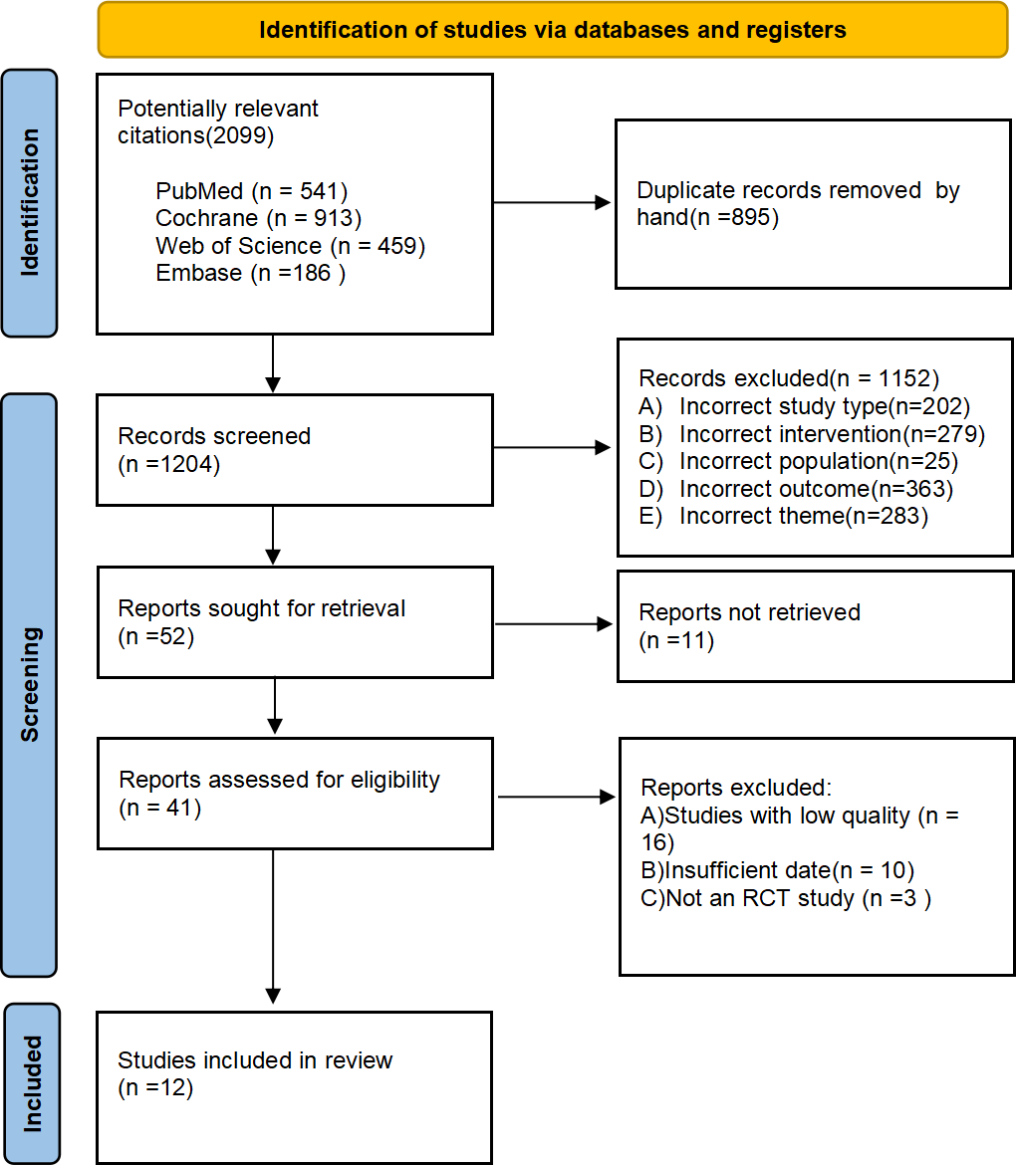
Prisma flow diagram of study selection process.

Our literature search identified 2,099 publications, the specific search strategy can be found in the attachment. After removing duplicates, 1,204 titles and abstracts were screened. Further, full-texts of 41 articles were read for a detailed evaluation. Finally, 12 articles were included in this systematic review and meta-analysis. Reasons for exclusion have been explained in the PRISMA flow-chart (Fig. 1).

### Characteristics of included studies

We systematically evaluated the 12 studies (10 double-blind RCTs and two open-label studies (Jou et al., 2008; Tranche et al., 2016)) that investigated the effects of soy isoflavones and related compounds on menopausal symptoms, with a focus on climacteric symptoms such as hot flashes and depressive symptoms. These studies encompassed various research designs, including randomized controlled trials and observational studies. The sample sizes in these studies ranged from 32 to 192 participants, involving over 1,000 participants in total. The geographical distribution was extensive, covering North America, South America, Asia, and Europe, with five studies conducted in Asia, which may reflect the application and effects of soy foods in different cultural contexts. All studies focused on postmenopausal women. Herein, seven studies specifically evaluated the impact of soy isoflavones on menopausal symptoms, whereas the other five studies explored the comprehensive effects of soy extracts on the quality of life, bone health, and cardiovascular health of postmenopausal women. Interventions included soy protein, soy isoflavone supplements, and soy beverages, with daily intervention doses ranging between 30 mg and 209 mg of soy isoflavones. The control group was usually administered a placebo. The intervention duration varied between 12 weeks and 2 years, providing a basis for assessing long-term effects. The primary outcomes included health-related quality of life, measured using instruments such as the Short Form-36 (SF-36) and MENQOL, climacteric symptoms assessed *via* the Kupperman Index and Greene Climacteric Scale, and depression symptoms assessed using the Zung Self-rating Depression Scale (ZSDS) and CES-D. The characteristics of the included studies are reported in Table 1.

**Table 1:** Characteristics of all trials included in the present meta-analysis.

Author	Design	Country	N randomized subjects (Soy isoflavones, comparison)	Age (range) (Soy isoflavones, comparison)	Exposure	Daily dosage	Other compounds	Duration of active treatment	Outcome (measures)
Atteritano et al. (2014)	Double-blind RCT, parallel	Italy	389 (198, 191 placebo)	(49–67) (53.00 ± 2.00, 52.00 ± 2.00)	Genistein	54 mg	Calcium carbonate (500 mg) Vitamin D (400 IU)	2 years	Zung Self-Rating Depression Scale Score^a^
Jou et al., 2008	Open label, parallel	China	64 (34, 30 placebo )	53.8 ± 3.8 EP group 53.6 ± 3.8 non-EP group 54.3 ± 2.8 placebo	Soy isoflavones	135 mg	None	6 months	Modified Kupperman Index^b^
Nourozi et al. (2015)	Double-blind RCT, parallel	Iran	80 (40, 40 placebo)	(45–60) (52.13 ± 3.05, 51.39 ± 2.89)	Soy isoflavones	185.55 mg	Calcium carbonate (500 mg) Vitamin D (200 IU)	8 months	MENQOL^c^
de Sousa-Munoz & Filizola (2009)	Double-blind RCT, parallel	Brazil	84 (42, 42 placebo)	(45–60) (53.35 ± 3.62)	Soy isoflavones	120 mg	None	16 weeks	CES-D^d^
Costa et al. (2017)	Double-blind RCT, parallel	Brazil	36 (19, 17 placebo)	(45–60) (56.0 ± 1.3, 52.7 ± 1.3)	Soy isoflavones	100 mg	None	10 weeks	Kupperman Index^e^ Menopause Rating Scale^f^ Cervantes Scale^g^
Frigo et al. (2022)	Double-blind RCT, parallel	Brazil	48 (24, 24 placebo)	(40–65) (51 ± 5.2, 50.7 ± 5.7)	Soy isoflavones	40.5 mg	Phytoestrogens derived from flaxseed (40.9 mg)	90 days	Kupperman Index
Secreto et al. (2004)	Double-blind RCT, parallel	Italy	117 (58, 59 placebo)	(≥35) (52.0, 52.0)	Soy isoflavones	80 mg	None	3 months	Greene Climacteric Scale^h^ Greene Psychological Subscale^i^
Tranche et al. (2016)	Open label, parallel	England	90 (45, 45 placebo)	(≥45) (51.8 ± 3.1, 51.5 ± 3.5)	Soy isoflavones	50 mg	None	12 weeks	Menopause Rating Scale
Evans et al. (2011)	Double-blind RCT, parallel	Canada	83 (41, 42 placebo)	(40–65) (53.39 ± 5.05, 53.50 ± 4.44)	Genistein	30 mg	None	12 weeks	Greene Climacteric Scale
Imhof et al. (2018)	Double-blind RCT, parallel	Austria Romania Germany	192 (97, 95 placebo)	(40–70) (54.3 ± 6.4, 53.6 ± 5.3)	Soy isoflavones	100 mg	None	12 weeks	Greene Climacteric Scale
Fontvieille, Dionne & Riesco (2017)	Double-blind RCT, parallel	Canada	31 (15, 16 placebo)	(50–70) (60.4 ± 3.4, 58.2 ± 5.7)	44 mg of daidzein 16 mg of glycitein 10 mg of genistein		None	12 months	SF-36^j^ Kupperman index PSS-10^k^
Na Takuathung et al. (2024)	Double-blind RCT, parallel	Thailand	100 (50, 50 placebo)	(48–65) (55.92 ± 3.52, 56.88 ± 3.62)	Soy isoflavones	209 mg	Grape seed extract Tomato extract (Lycopersicon esculentum Mill) Vitamins Minerals	12 weeks	Modified Kupperman Index MENQOL

**Notes.**

a This is a self-reporting scale consisting of 20 items to assess the presence and severity of depressive symptoms.

b An adaptation of the original Kupperman Index, used to assess the severity of menopausal symptoms.

c The Menopause-Specific Quality of Life questionnaire is a tool designed to measure the impact of menopause on a woman’s quality of life.

d The Center for Epidemiologic Studies Depression Scale is a self-reporting questionnaire used to measure symptoms of depression in the general population.

e A widely used scale to assess the severity of menopausal symptoms, which was one of the first tools developed for this purpose.

f A validated instrument used to evaluate the severity of climacteric symptoms and the effectiveness of treatments for menopausal symptoms.

g A 31- item questionnaire used to assess the health-related quality of life in women during the menopausal transition.

h A self-assessment tool consisting of 21 items across five dimensions to evaluate menopausal symptoms, including anxiety, depression, somatic symptoms, vasomotor symptoms, and sexual function.

i A part of the Greene Climacteric Scale that focuses on psychological symptoms such as anxiety and depression.

j A multipurpose, short-form health survey with 36 questions about perceptions of health and well-being.

k The Perceived Stress Scale-10 is a self-report measure developed to assess the global level of perceived stress.

### Risk of bias assessment

**Figure 2:**
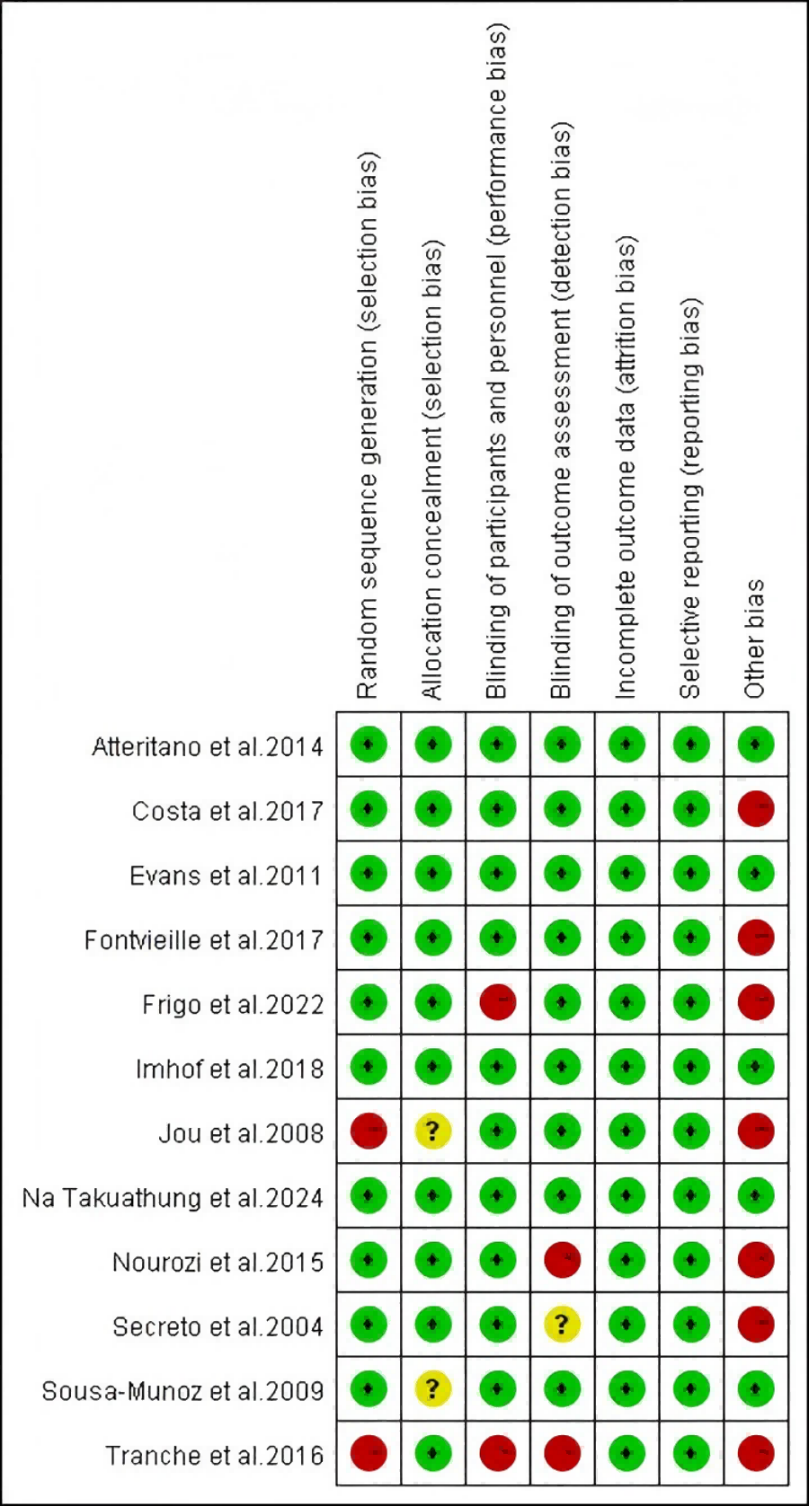
Risk of bias assessment of the included studies. Notes: Atteritano et al., 2014, Costa et al., 2017, Evans et al., 2011, Fontvieille, Dionne & Riesco, 2017, Frigo et al., 2022, Imhof et al., 2018, Jou et al., 2008, Na Takuathung et al., 2024, Nourozi et al., 2015, Secreto et al., 2004, de Sousa-Munoz & Filizola, 2009, Tranche et al. (2016).

**Figure 3:**
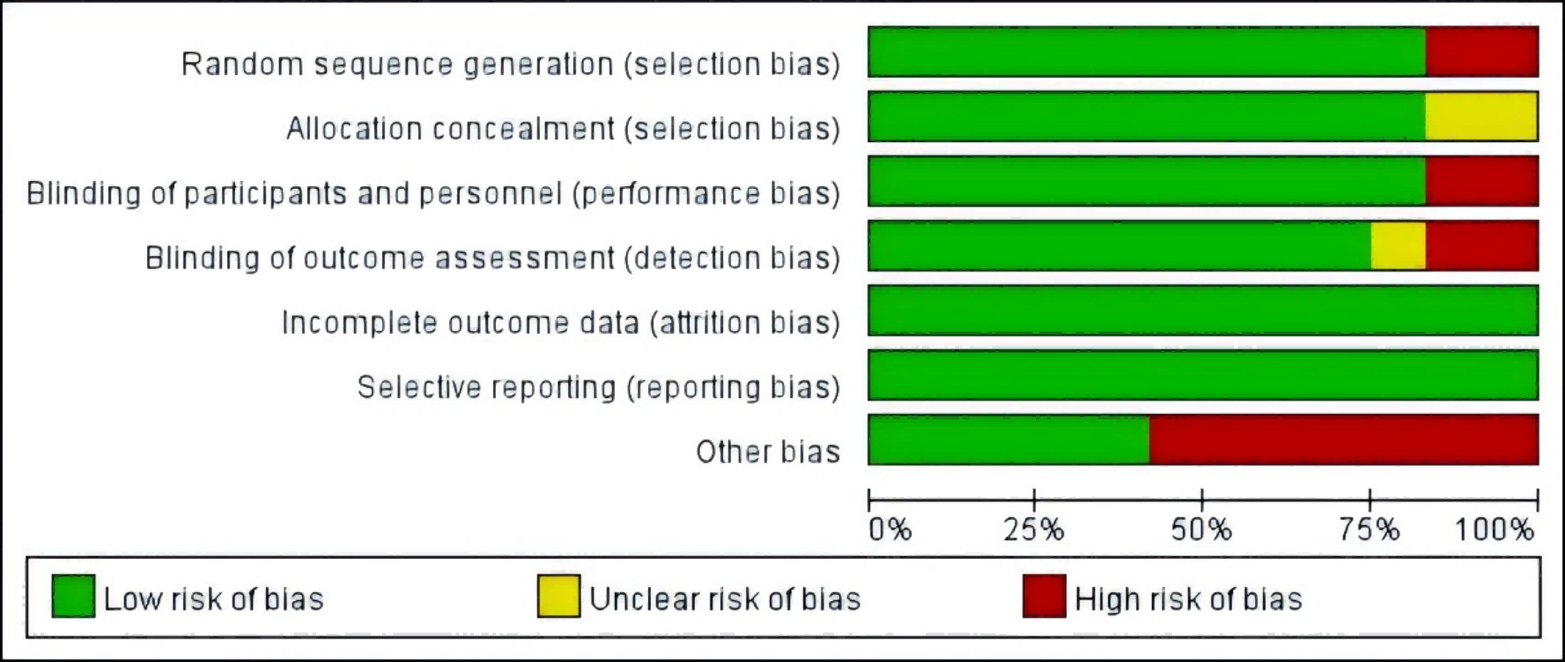
Risk of bias assessment of the included studies.

The included studies were assessed for risk of bias using the Cochrane Risk of Bias Tool by two independent reviewers, Fan and Lin, with discrepancies resolved through discussion with Guo. In random sequence generation, the studies by Jou et al. (2008) and Tranche et al. (2016) were rated as high risk owing to their open-label design. In allocation concealment, studies by Jou et al. (2008) and de Sousa-Munoz & Filizola (2009) were rated as unclear owing to lack of allocation details. In blinding of participants and personnel, studies by Frigo et al. (2022) and Tranche et al. (2016) were rated high risk because researchers knew the group assignments. In blinding of outcome assessment, the study Secreto et al. (2004) was rated unclear owing to missing information, whereas those by Nourozi et al. (2015) and Tranche et al. (2016) were rated high risk owing to researchers’ knowledge of assignments. In other bias evaluation, studies by Costa et al. (2017) and Fontvieille, Dionne & Riesco (2017) were rated high risk owing to the inclusion of non-pharmacological interventions (exercise); those by Frigo et al. (2022), Secreto et al. (2004), and Nourozi et al. (2015) were rated high risk owing to the inclusion of additional substances (*e.g.*, melatonin, calcium-D); and those by Jou et al. (2008) and Tranche et al. (2016) were rated high risk owing to potential placebo effects in open-label studies. The studies by Atteritano et al. (2014), Evans et al. (2011), Imhof et al. (2018), and Na Takuathung et al. (2024) showed no relevant bias risks. The risk of bias in the included studies is presented in Figs. 2 and 3.

### Effect of soy isoflavones on menopausal symptoms

**Figure 4:**
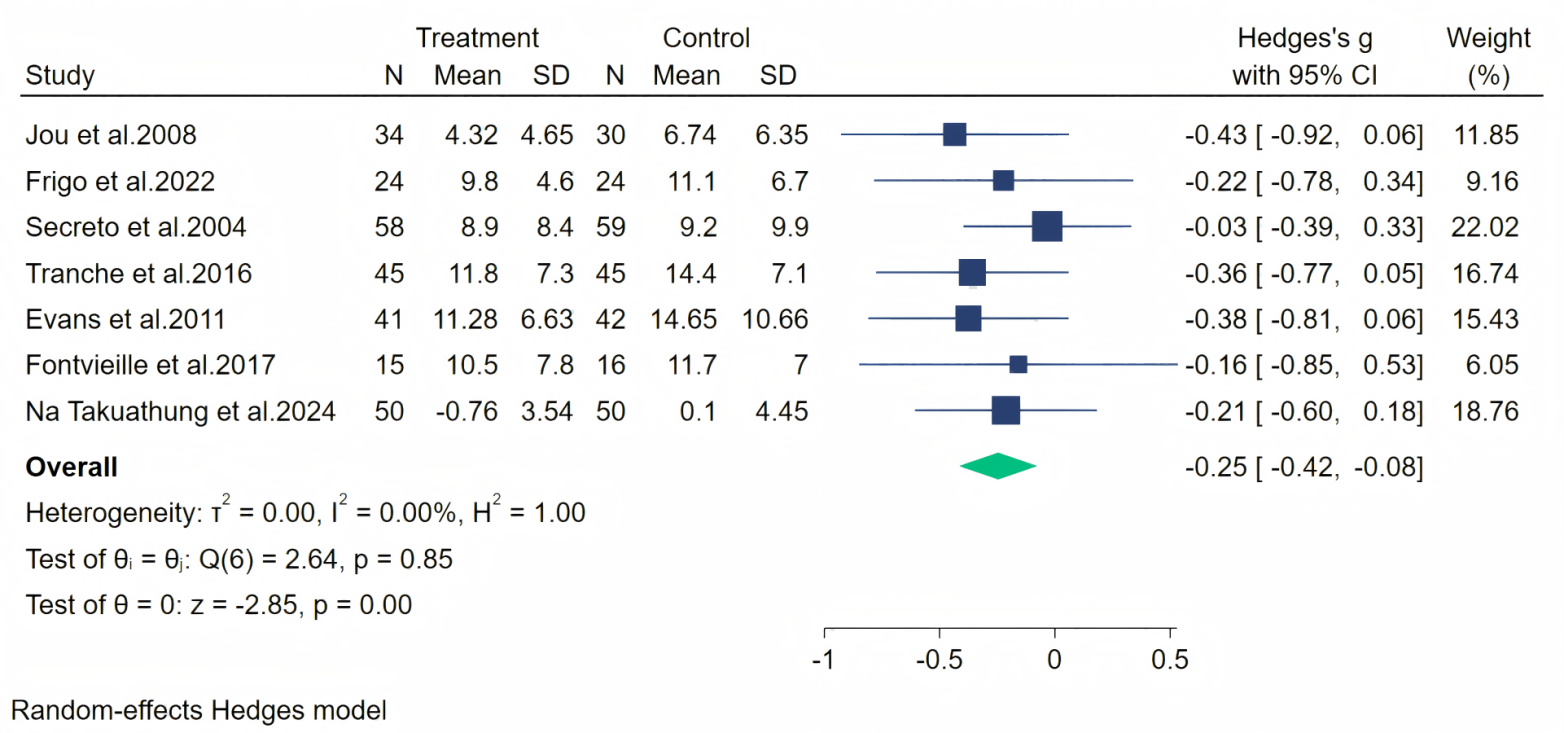
Effect of soy isoflavones on menopausal symptoms. Notes: Jou et al., 2008, Frigo et al., 2022, Secreto et al., 2004, Tranche et al. (2016), Evans et al., 2011, Fontvieille, Dionne & Riesco, 2017, Na Takuathung et al., 2024.

**Figure 5:**
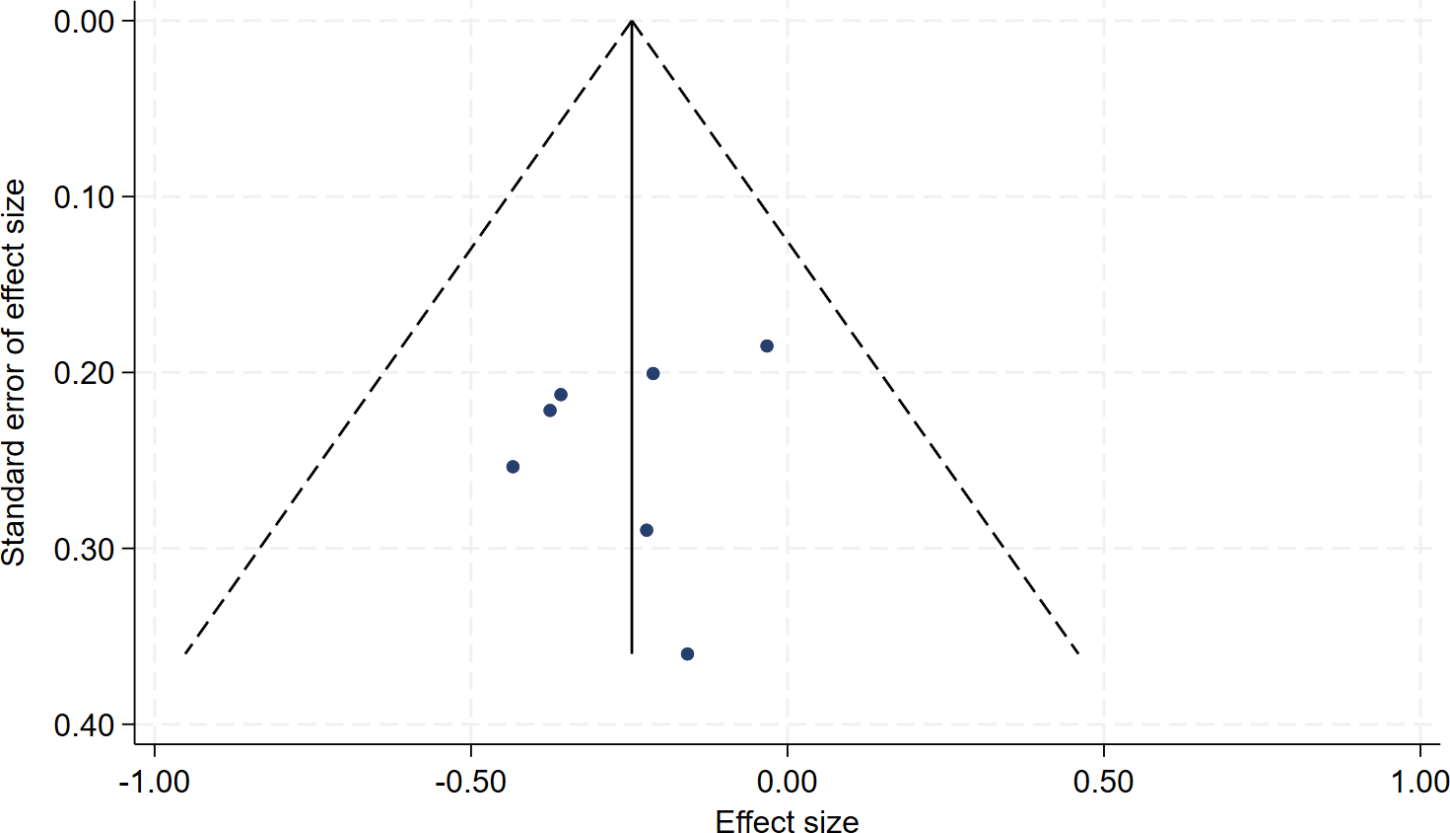
Funnel plot of bias analysis.

**Figure 6:**
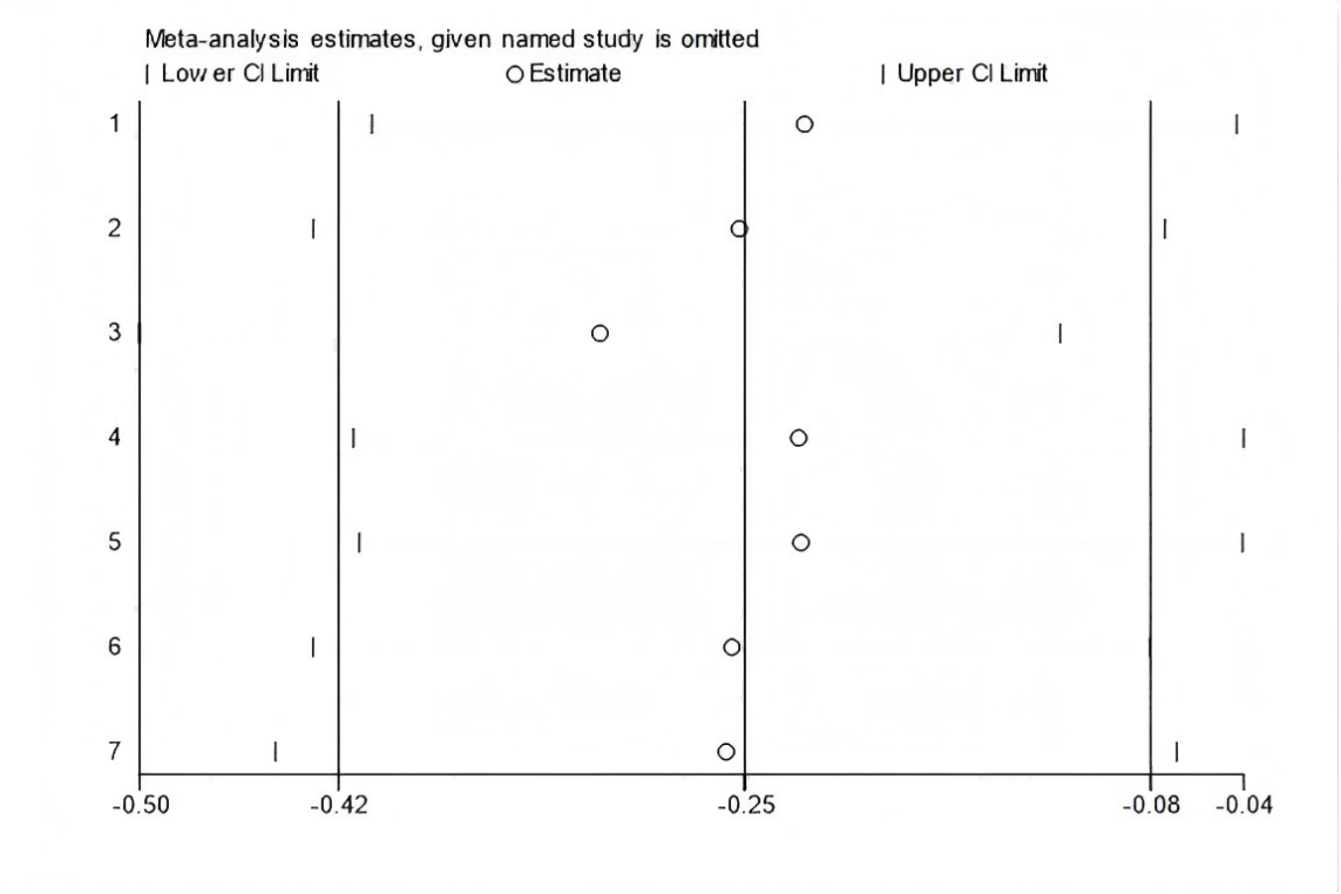
Sensitivity analysis.

In all, seven studies measured menopausal symptoms, which represent the primary outcome of this systematic review. As shown in Table 1, the measurement of menopausal symptoms was performed using various scales, such as the Kupperman Index (KI) (Tao et al., 2013), Modified Kupperman Index (MKI) (Tao et al., 2013), Menopause Rating Scale (MRS) (Dinger et al., 2006), and Greene Climacteric Scale (GCS) (Greene, 1998). Random-effects meta-analysis (seven studies, 533 participants, with 267 in the soy isoflavones group and 266 in the control group) revealed that soy isoflavones have a certain therapeutic effect on menopausal symptoms (Hedges’ g = −0.25, 95% CI [−0.42 to −0.08], *p* = 0.00), with a moderate effect size and low heterogeneity (*I*^2^ = 0.00%). After removing each study from the overall effect size, no significant differences were found. The forest plot is presented in Fig. 4. The bias analysis is shown in Fig. 5 and the sensitivity analysis is shown in Fig. 6.

**Figure 7:**
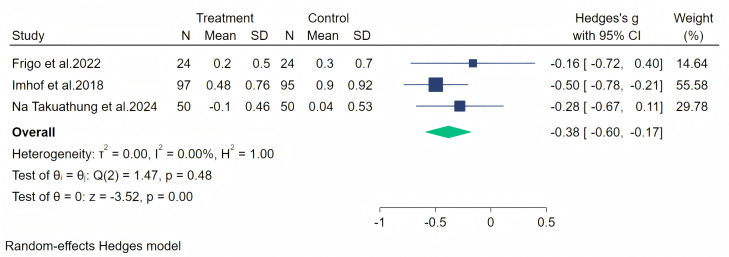
Effect of soy isoflavones on headache symptoms. Notes: Na Takuathung et al., 2024, Imhof et al. (2018), Frigo et al., 2022.

Headache symptoms were measured through three studies, and a random-effects meta-analysis (three studies, 340 participants, 171 in the soy isoflavones group, and 169 in the control group) showed that soy isoflavones have some therapeutic effect on headaches, with a moderate effect size (Hedges’ g = −0.38, 95% CI [−0.60 to −0.17], *p* = 0.00). The heterogeneity between studies was low (*I*^2^ = 0.00%). On removing each study and analyzing the overall effect size, we found no significant difference in the results. The meta-analysis is presented in Fig. 7.

**Figure 8:**
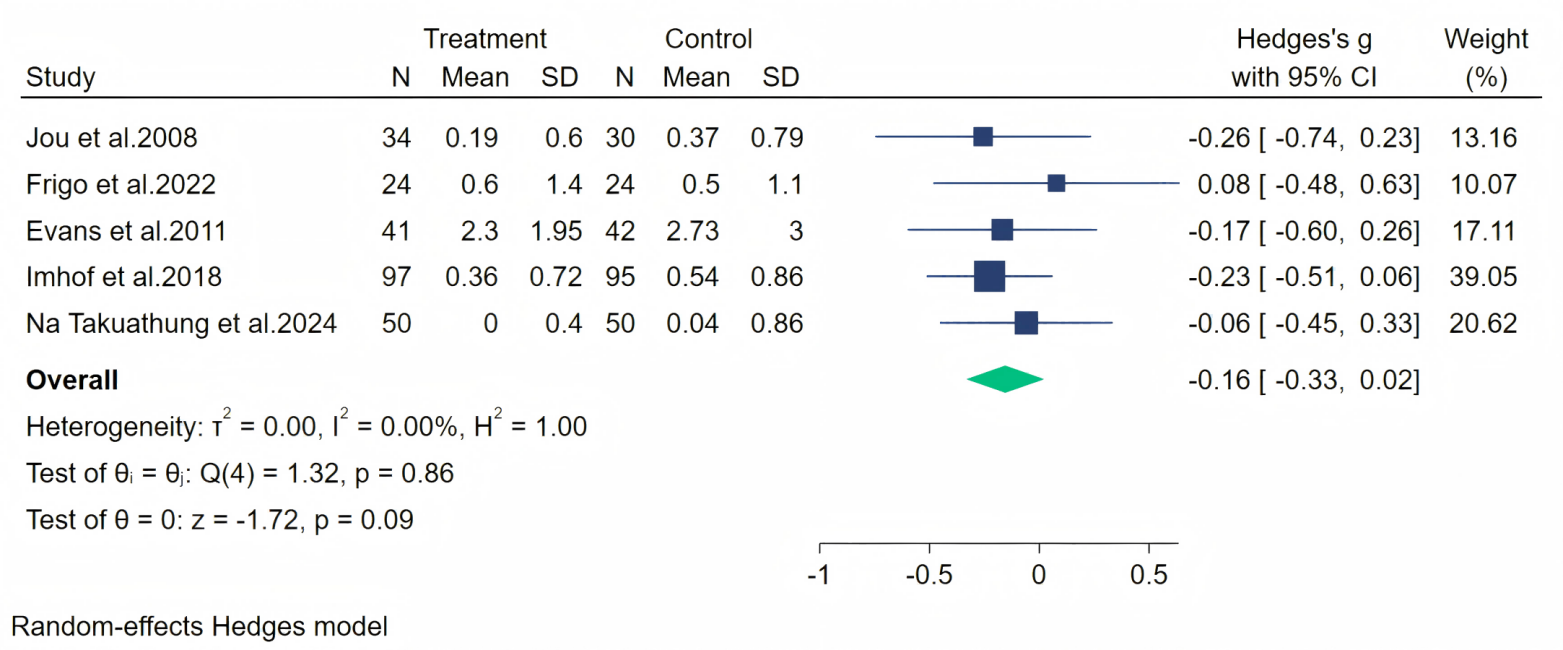
Effect of soy isoflavones on paresthesia symptoms. Notes: Jou et al., 2008, Frigo et al., 2022, Evans et al., 2011, Imhof et al., 2018, Na Takuathung et al., 2024.

Paresthesia symptoms were measured through five studies, and a random-effects meta-analysis (five studies, 487 participants, with 246 in the soy isoflavone group and 241 in the control group) indicated that soy isoflavones had no significant effect in the treatment of Paresthesia symptoms, with a low effect size (Hedges’ g = −0.16, 95% CI [−0.33 to 0.22], *p* = 0.09) and low heterogeneity among studies (*I*^2^ = 0.00%). On removing each study and analyzing the overall effect size, we did not find significant differences in the results. The meta-analysis is presented in Fig. 8.

**Figure 9:**
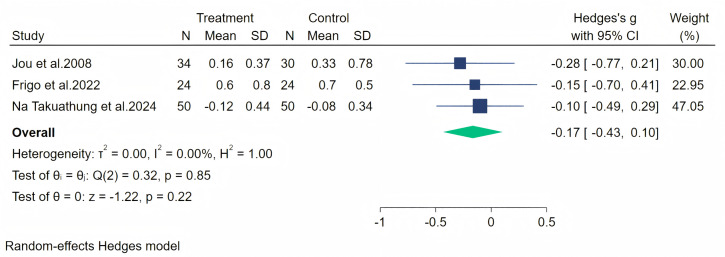
Effect of soy isoflavones on fatigue symptoms. Notes: Na Takuathung et al., 2024, Frigo et al., 2022, Jou et al., 2008.

Fatigue symptoms were measured across four studies, and the first data analysis showed high heterogeneity (*I*^2^ = 82.54%, *p* = 0.08). After evaluation, we considered that the heterogeneity originated from the article by Imhof et al. (2018) (owing to significant baseline differences), and therefore, we excluded this article from our analysis. The random-effects meta-analysis (including three studies, 212 participants: 108 in the soy isoflavone group and 104 in the control group) indicated that soy isoflavones were not significantly effective in treating fatigue symptoms, with a low effect size (Hedges’ g = −0.17, 95% CI [−0.43 to 0.10], *p* = 0.22) and heterogeneity between studies (*I*^2^ = 0.00%). Meta-analysis is presented in Fig. 9.

**Figure 10:**
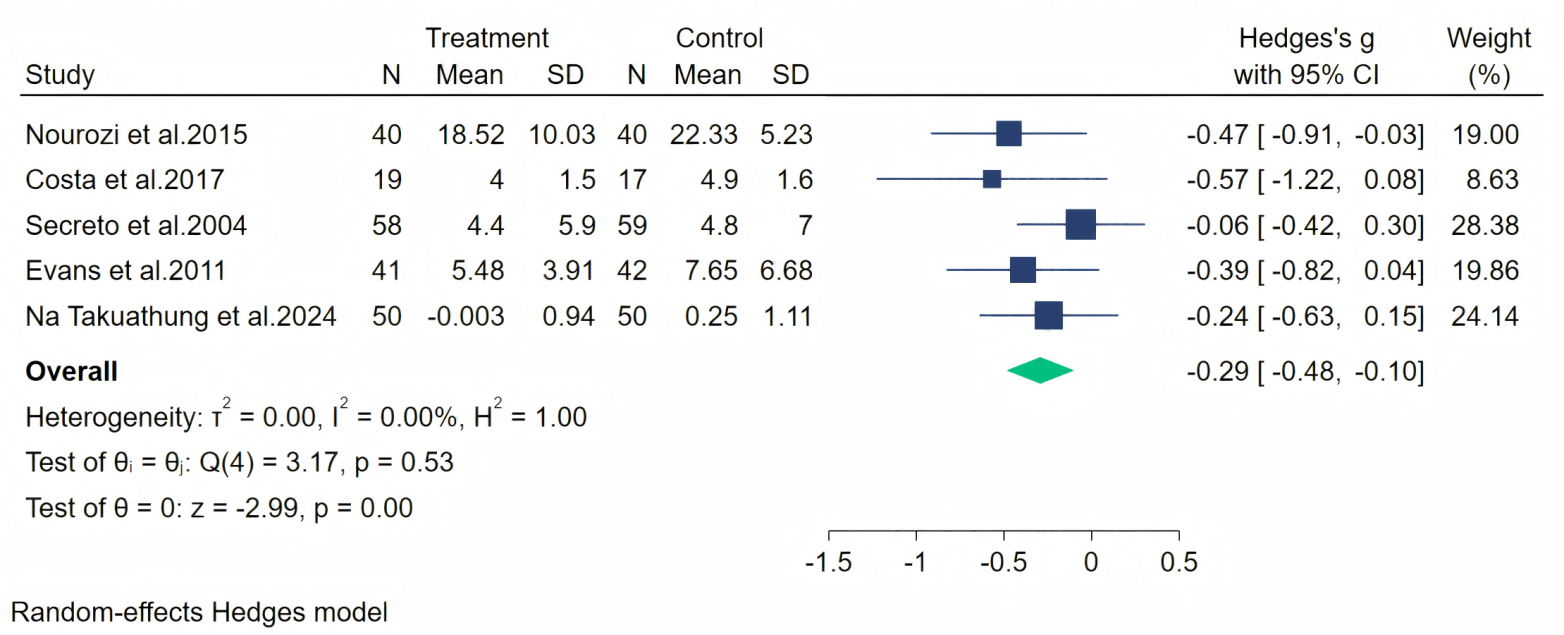
Effect of soy isoflavones on psychosocial symptoms. Notes: Nourozi et al., 2015, Costa et al., 2017, Secreto et al., 2004, Evans et al., 2011, Na Takuathung et al. (2024).

The psychosocial symptoms were measured through five studies. A random-effects meta-analysis (five studies, 416 participants: 208 in the soy isoflavone group and 208 in the control group) indicated that soy isoflavones have a moderate effect on psychosocial symptoms, with a medium effect size (Hedges’ g = −0.29, 95% CI [−0.48 to −0.10], *p* = 0.00) and low heterogeneity between studies (*I*^2^ = 0.00%). By removing each study and analyzing the overall effect size, we found no significant difference in the results. Meta-analysis is presented in Fig. 10.

**Figure 11:**
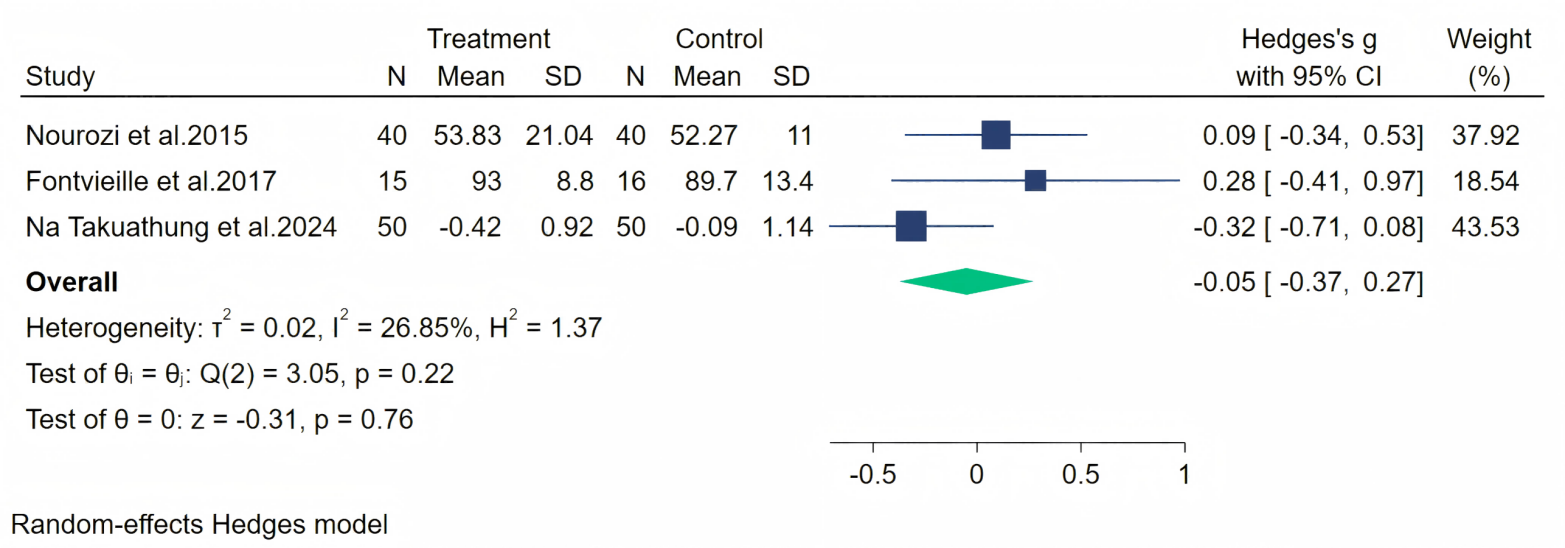
Effect of soy isoflavones on physical symptoms. Notes: Na Takuathung et al., 2024, Nourozi et al., 2015, Fontvieille, Dionne & Riesco, 2017.

Subsequently, physical symptoms were measured through three studies, and a random-effects meta-analysis (three studies, 211 participants: 105 in the soy isoflavone group and 106 in the control group) indicated that soy isoflavones have no therapeutic effect on physical symptoms, with a low effect size (Hedges’ g = −0.05, 95% CI [−0.37 to 0.27], *p* = 0.76) and moderate heterogeneity between studies (*I*^2^ = 26.85%). By removing each study and analyzing the overall effect size, no significant difference was observed in the results. Meta-analysis is presented in Fig. 11.

**Figure 12:**
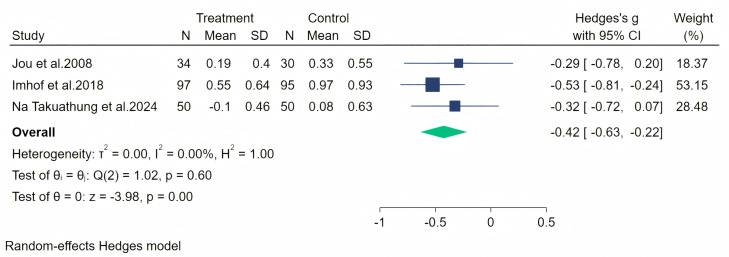
Effect of soy isoflavones on palpitation symptoms. Notes: Jou et al., 2008, Imhof et al., 2018, Na Takuathung et al., 2024.

Palpitation symptoms were measured through three studies, and a random-effects meta-analysis (three studies, 356 participants: 181 in the soy isoflavone group and 175 in the control group) indicated that soy isoflavones have a moderate effect on alleviating palpitation symptoms (Hedges’ g = −0.42, 95% CI [−0.63 to −0.22], *p* = 0.00). The heterogeneity between studies was low (*I*^2^ = 0.00%). By removing each study and analyzing the overall effect size, we found no significant difference in the results. Meta-analysis is presented in Fig. 12.

**Figure 13:**
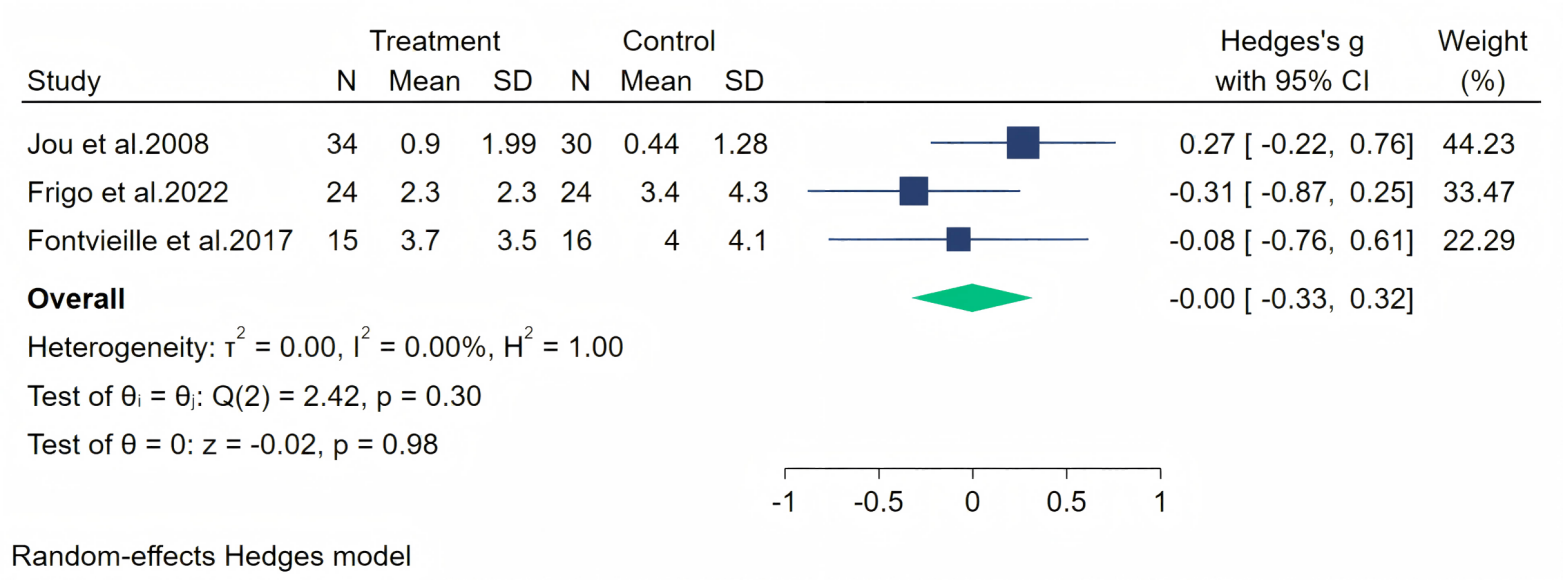
Effect of soy isoflavones on hot flashes symptoms. Notes: Jou et al., 2008, Imhof et al., 2018, Fontvieille, Dionne & Riesco, 2017.

Hot flashes were measured across four studies, and the initial analysis showed high heterogeneity (*I*^2^ = 86.67%, *p* = 0.25). Upon evaluation, we considered that the heterogeneity stemmed from the study by Imhof et al. (2018) (owing to significant baseline differences), and therefore, we excluded this article from our analysis. The random-effects meta-analysis (three studies, 143 participants: 73 in the soy isoflavone group and 70 in the control group) indicated that soy isoflavones are not effective in alleviating hot flashes, with a low effect size (Hedges’ g = −0.00, 95% CI [−0.33 to 0.32], *p* = 0.98) and low heterogeneity between studies (*I*^2^ = 0.00%). Meta-analysis is presented in Fig. 13.

**Figure 14:**
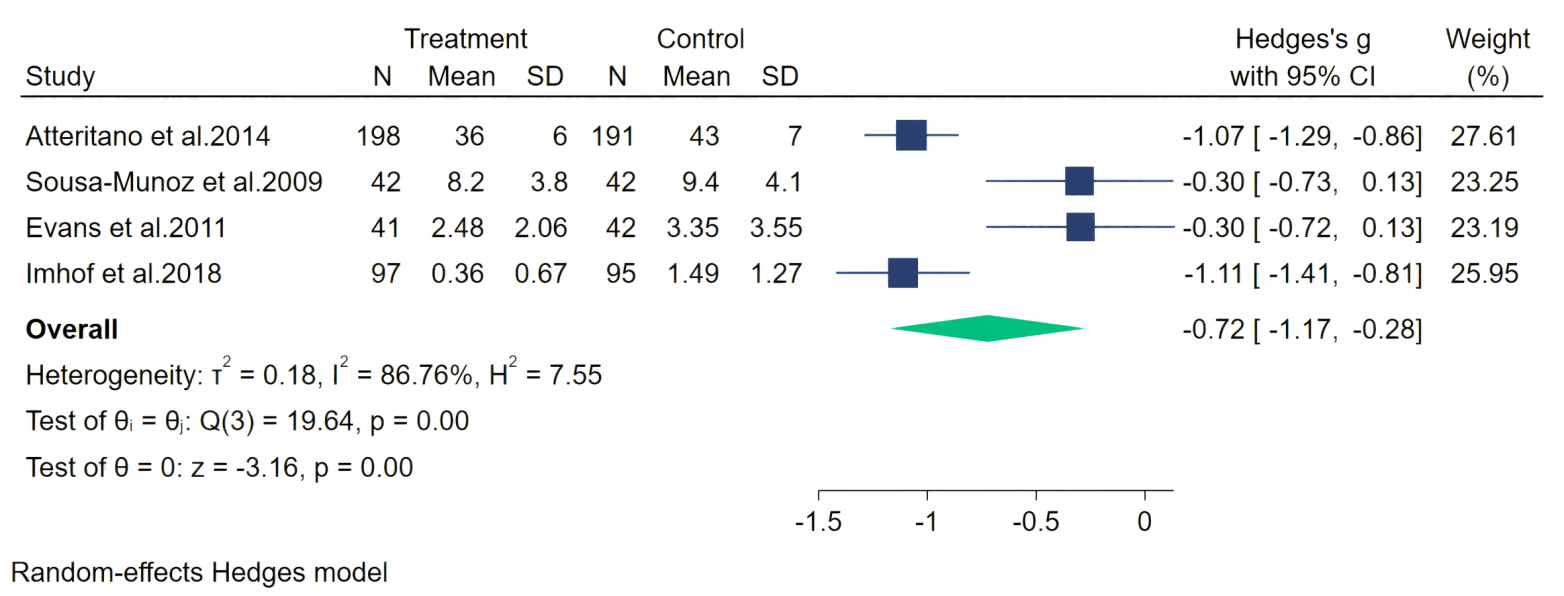
Effect of soy isoflavones on depression symptoms. Notes: Atteritano et al., 2014, de Sousa-Munoz & Filizola, 2009, Evans et al., 2011, Imhof et al., 2018.

Furthermore, depression symptoms were measured through four studies. A random-effects meta-analysis (four studies, 748 participants: 378 in the soy isoflavone group and 370 in the control group) showed a significant therapeutic effect of soy isoflavones on depression symptoms, with a high effect size (Hedges’ g = −0.72, 95% CI [−1.17 to −0.28], *p* = 0.00). However, the heterogeneity between studies was high (*I*^2^ = 86.76%). By removing the influence of each study on the overall effect size, no significant differences were found in the results. Meta-analysis is presented in Fig. 14. Considering the high heterogeneity and the limited number of included studies, we conducted meta-regression analyses separately for duration, dose, and population. The specific results are shown in Table 2.

**Table 2:** Meta-regression of depression symptom.

Meta-regression of depression		
Variate	Estimate, 95% CI	*p*-value
Duration	−1.59 [−1.93 to 0.89]	0.254
Dose	−1.03 [−3.53 to 2.16]	0.411
Population	−0.77 [−1.63 to 1.14]	0.523
Baseline symptom severity	−1.50 [−2.33 to 1.12]	0.271

**Figure 15:**
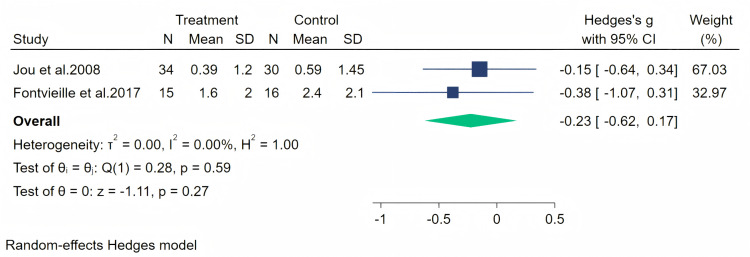
Effect of soy isoflavones on excessive sweating symptoms.

Excessive sweating as a perimenopausal symptom was measured through two studies, and a random-effects meta-analysis (two studies, 95 participants: 49 in the soy isoflavone group and 46 in the control group) indicated that soy isoflavones were not significantly effective for excessive sweating symptoms, with a low effect size (Hedges’ g = −0.23, 95% CI [−0.62 to 0.17], *p* = 0.27) and low heterogeneity between studies (*I*^2^ = 0.00%). By removing the influence of each study on the overall effect size, no significant differences were found in the results. Meta-analysis is presented in Fig. 15.

**Figure 16:**
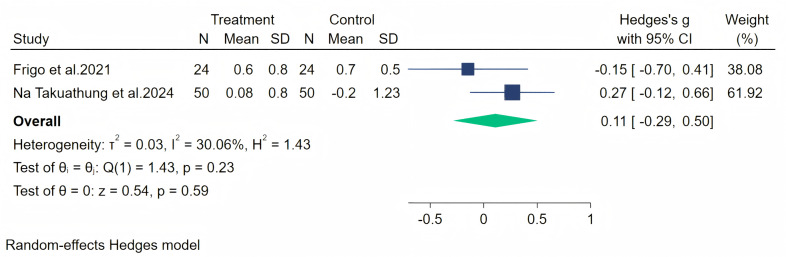
Effect of soy isoflavones on insomnia symptoms. Notes: Frigo et al., 2022, Na Takuathung et al., 2024.

Insomnia symptoms were measured through two studies, and a random-effects meta-analysis (two studies, 148 participants: 74 in the soy isoflavone group and 74 in the control group) indicated that soy isoflavones have no significant effect on insomnia, with a low effect size (Hedges’ *g* = 0.11, 95% CI [−0.29 to 0.50], *p* = 0.59) and moderate heterogeneity between studies (*I*^2^ = 30.06%). By removing the influence of each study on the overall effect size, no significant differences were found in the results. Meta-analysis is presented in Fig. 16.

**Figure 17:**
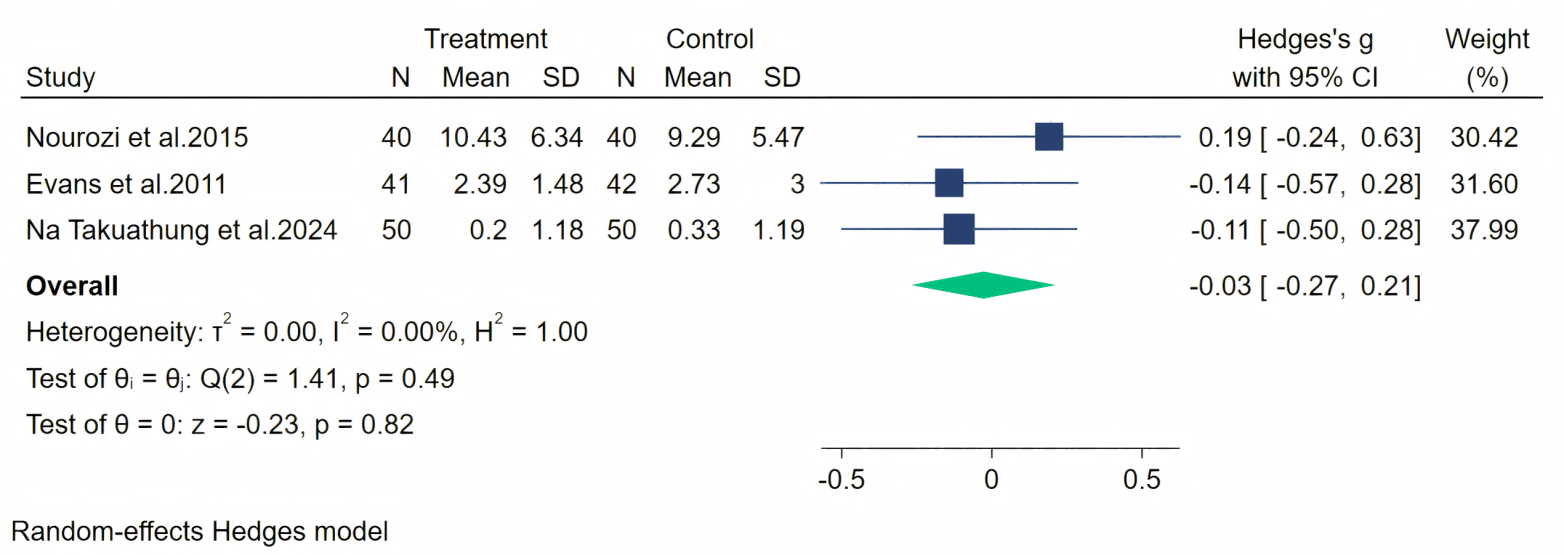
Effect of soy isoflavones on vasomotor. Notes: Nourozi et al., 2015, Evans et al., 2011, Na Takuathung et al., 2024.

Vasomotor symptoms were measured through three studies, and a random-effects meta-analysis (three studies, 263 participants: 131 in the soy isoflavone group and 132 in the control group) indicated that soy isoflavones are not significantly effective in treating vasomotor symptoms, with a low effect size (Hedges’ g = −0.03, 95% CI [−0.27 to 0.21], *p* = 0.82). Heterogeneity between studies was low (*I*^2^ = 0.00%). By removing the influence of each study on the overall effect size, we did not find any significant differences in the results. Meta-analysis is presented in Fig. 17.

## Discussion

Soy isoflavones, including genistein, daidzein, and glycitein, are recognized for their multifaceted pharmacological actions, particularly with regard to anti-inflammation, antioxidant activity, and menopausal symptom alleviation. These compounds exhibit anti-inflammatory effects by modulating key signaling pathways and reducing the release of inflammatory mediators, which are crucial for managing inflammation-related menopausal symptoms. Their antioxidant properties are highlighted by their ability to scavenge reactive oxygen species (ROS), thereby protecting against oxidative damage and the aging process. Furthermore, soy isoflavones have been shown to alleviate menopausal symptoms by acting as phytoestrogens, binding to estrogen receptors and exerting both agonistic and antagonistic effects, which can help reduce and alter vasomotor symptoms. Recent research has also underscored their potential in preventing bone loss (Akhlaghi et al., 2020), reducing cancer risk (Fan et al., 2022), and exhibiting anti-obesity and anti-diabetes effects (Dwivedi et al., 2024). These findings position soy isoflavones as key bioactive compounds in the management of menopause and promotion of women’s health.

Our research findings indicate that soy isoflavones have a certain positive effect on menopausal symptoms, which are very commonly observed in perimenopausal women. In particular, the Hedges’ g value, indicating the overall effect size for menopausal symptoms, was −0.25 (95% CI [−0.42 to −0.08], *p* = 0.00), indicating a small but statistically significant reduction in symptom severity. However, this effect size is considered small according to Cohen’s criteria (Gignac & Szodorai, 2016), and its clinical significance remains uncertain. For example, although a reduction in symptoms was observed, the magnitude of this reduction may not be sufficient to result in substantial improvements in quality of life for all individuals. Similarly, the effect size was also small for psychosocial symptoms (Hedges’ g = −0.29), suggesting that while soy isoflavones may provide some benefit, the impact may be limited in a clinical context. The effect size was larger for depression symptoms (Hedges’ g = −0.72), indicating a potentially more meaningful clinical benefit, although this finding was accompanied by high heterogeneity among studies (*I*^2^ = 86.76%). This indicates that although soy isoflavones may be effective for some individuals, the variability in response may limit their broad applicability.

Soy isoflavones appear to modulate the NF-κB signaling pathway, a critical mediator in inflammatory and immune responses (Ma et al., 2021). This pathway modulation can be beneficial in reducing chronic inflammation, thereby potentially reducing the risks associated with inflammatory diseases. Furthermore, the interaction of soy isoflavones with the JAK-STAT pathway, important in cellular processes such as growth and apoptosis, suggests their role in cancer management (Wu et al., 2021). Soy isoflavones can help inhibit undesirable cell proliferation and metastasis by influencing this pathway, thus offering a complementary approach to traditional cancer therapies. Skin health may also benefit from the inclusion of soy isoflavones in the diet or skincare regimen. Their ability to counteract UV-induced damage by promoting DNA repair and reducing the expression of inflammatory genes such as GADD45 and COX-2 is significant (Ou et al., 2021). Such protective and reparative actions not only help in preventing skin cancer but also combat premature aging. Enhanced collagen synthesis and reduced inflammatory cytokines contribute to improved skin elasticity and reduced wrinkle formation, manifesting as visible anti-aging effects. Moreover, PI3K-Akt signaling pathway regulation by soy isoflavones highlights their role in cell survival and proliferation (Khezri et al., 2022). The involvement of this pathway in cancer cell growth and survival underscores the potential of soy isoflavones in influencing tumor dynamics, possibly offering a natural adjunct therapy for cancer management. Potential interaction with drug metabolism, particularly through cytochrome P450 enzyme modulation, indicates that the clinical use of soy isoflavones deserves critical consideration (Ronis, 2016). As these enzymes play a significant role in the metabolism of many drugs, soy isoflavones can alter the pharmacokinetics of concurrent medications, necessitating careful management and monitoring by healthcare providers.

Our research findings indicate that soy isoflavones have a positive effect on menopausal symptoms, which are very common in perimenopausal women. This finding contradicts a recent study (Gencturk, Bilgic & Kaban, 2024), reporting that soy isoflavones have no effect on menopausal symptoms (including vasomotor, psychosocial, physical, sexual, and urogenital discomfort) or quality of life in postmenopausal women. However, we found that soy isoflavones have a significant therapeutic effect on depression symptoms. We consider that the discrepancy may arise from differences in search term settings and inclusion criteria, which might have led to a relatively small number of studies being included and potentially biased the results.

This study was limited to only 12 articles, with the evaluation of menopausal symptoms conducted in only seven trials. To better understand the potential benefits of soy isoflavones as a complementary treatment for menopausal symptoms, further research is necessary. Another significant limitation concerns the patient population; all included studies typically featured small sample sizes. Given the accessibility and favorable tolerance of soy isoflavones among patients, future studies should aim to include larger cohorts. Moreover, the inability to access databases for all trials hindered our capacity to conduct an individual patient data analysis, which would have provided a more thorough evaluation. It is important to highlight that most studies were conducted in European countries, where soy consumption is relatively low. Investigating the effects of soy isoflavones in Asian countries, where soy foods are more commonly consumed, would be an intriguing direction for future research. It is also crucial to point out that the primary reason soy isoflavones are generally not recommended for breast cancer patients is their potential for bidirectional hormonal modulation and their effects on estrogen receptors. Given their structural resemblance to estrogen, soy isoflavones can bind to estrogen receptors and produce similar effects. In breast cancer patients, particularly those with estrogen receptor-positive (ER+) tumors, soy isoflavones may imitate estrogen’s actions and promote the proliferation of breast cancer cells (Yamashita et al., 2022). Moreover, certain studies suggest that at lower concentrations, soy isoflavones may not inhibit but actually stimulate the growth of estrogen-dependent breast cancer cells, such as MCF-7 (Uifalean et al., 2015). Other research has indicated a dose-dependent relationship, wherein increased levels of soy isoflavones from soy protein are associated with enhanced growth of estrogen-dependent tumor cells (Johnson et al., 2016). Continued investigation is vital to clarify these contentious findings.

Adverse effects and contraindications of soy isoflavones should be carefully considered in clinical practice. With regard to adverse effects, some individuals may experience gastrointestinal discomfort such as nausea, abdominal pain, or diarrhea after consuming soy isoflavones (Al-Nakkash & Kubinski, 2020). Allergic reactions, including skin itching, redness, and rashes, may also occur among individuals allergic to soy products (Wang, He & Raghavan, 2023). In addition, high doses of soy isoflavones potentially affect liver, kidney, and reproductive system functions in animal models (Neshatbini Tehrani et al., 2024). However, these findings have not been conclusively translated to humans. Regarding contraindications, soy isoflavones should be avoided by individuals with a history of estrogen-related diseases, such as breast hyperplasia, breast cancer, and uterine cancer, as they may exacerbate these conditions. The currently recommended intake of soy isoflavones for perimenopausal women is mostly in the range of 50–100 mg per day (Kang et al., 2022; Khapre, Deshmukh & Jain, 2022). Prepubescent girls, pregnant and breastfeeding women, and individuals with hypothyroidism should also refrain from using soy isoflavones owing to the potential adverse effects on growth, development, and thyroid function (Huser et al., 2018). Furthermore, patients administering anti-estrogen medications should avoid soy isoflavones to prevent interference with their treatment. In clinical practice, it is essential to weigh the potential benefits of soy isoflavones against the risks and to closely monitor patients for any adverse reactions.

In conclusion, our study suggests that soy isoflavones may have some potential benefits in relieving certain menopausal symptoms, particularly depressive symptoms. However, the small effect sizes for many outcomes, wide confidence intervals, and methodological inconsistencies among the included studies limit the certainty of these findings. The clinical significance of these results remains uncertain, and further research is needed to better understand the potential benefits and risks of soy isoflavones for the management of menopausal symptoms. Future studies should focus on larger sample sizes, standardized intervention protocols, and the inclusion of diverse populations to provide more robust evidence. In addition, investigating the potential adverse effects and contraindications of soy isoflavones is important to inform clinical practice and ensure patient safety.

##  Supplemental Information

10.7717/peerj.19715/supp-1 Supplemental Information 1 PRISMA checklist

10.7717/peerj.19715/supp-2 Supplemental Information 2 Search strategy

10.7717/peerj.19715/supp-3 Supplemental Information 3 Characteristics of all trials included in the present meta-analysis

10.7717/peerj.19715/supp-4 Supplemental Information 4 Audience

## Additional Information and Declarations

### Data Availability

The following information was supplied regarding data availability: This is a systematic review/meta-analysis.

## References

[ref-1] Akhlaghi M, Ghasemi Nasab M, Riasatian M, Sadeghi F (2020). Soy isoflavones prevent bone resorption and loss, a systematic review and meta-analysis of randomized controlled trials. Critical Reviews in Food Science and Nutrition.

[ref-2] Al-Nakkash L, Kubinski A (2020). Soy isoflavones and gastrointestinal health. Current Nutrition Reports.

[ref-3] Atteritano M, Mazzaferro S, Bitto A, Cannata ML, D’Anna R, Squadrito F, Macri I, Frisina A, Frisina N, Bagnato G (2014). Genistein effects on quality of life and depression symptoms in osteopenic postmenopausal women: a 2-year randomized, double-blind, controlled study. Osteoporosis International.

[ref-4] Avis NE, Stellato R, Crawford S, Bromberger J, Ganz P, Cain V, Kagawa-Singer M (2001). Is there a menopausal syndrome? Menopausal status and symptoms across racial/ethnic groups. Social Science and Medicine.

[ref-5] Cagnacci A, Venier M (2019). The controversial history of hormone replacement therapy. Medicina.

[ref-6] Chen LR, Ko NY, Chen KH (2019). Isoflavone supplements for menopausal women: a systematic review. Nutrients.

[ref-7] Chen MN, Lin CC, Liu CF (2015). Efficacy of phytoestrogens for menopausal symptoms: a meta-analysis and systematic review. Climacteric.

[ref-8] Costa JG, Giolo JS, Mariano IM, Batista JP, Ribeiro ALA, Souza TCF, De Oliveira EP, Resende APM, Puga GM (2017). Combined exercise training reduces climacteric symptoms without the additive effects of isoflavone supplementation: a clinical, controlled, randomised, double-blind study. Nutrition and Health.

[ref-9] de Sousa-Munoz RL, Filizola RG (2009). Efficacy of soy isoflavones for depressive symptoms of the climacteric syndrome. Maturitas.

[ref-10] Dinger J, Zimmermann T, Heinemann LA, Stoehr D (2006). Quality of life and hormone use: new validation results of MRS scale. Health and Quality of Life Outcomes.

[ref-11] Dwivedi S, Singh V, Sharma K, Sliti A, Baunthiyal M, Shin JH (2024). Significance of soy-based fermented food and their bioactive compounds against obesity, diabetes, and cardiovascular diseases. Plant Foods for Human Nutrition.

[ref-12] Evans M, Elliott JG, Sharma P, Berman R, Guthrie N (2011). The effect of synthetic genistein on menopause symptom management in healthy postmenopausal women: a multi-center, randomized, placebo-controlled study. Maturitas.

[ref-13] Fan Y, Wang M, Li Z, Jiang H, Shi J, Shi X, Liu S, Zhao J, Kong L, Zhang W, Ma L (2022). Intake of soy, soy isoflavones and soy protein and risk of cancer incidence and mortality. Frontiers in Nutrition.

[ref-14] Fontvieille A, Dionne IJ, Riesco E (2017). Long-term exercise training and soy isoflavones to improve quality of life and climacteric symptoms. Climacteric.

[ref-15] Frigo M, de Barros E, Dos Santos PCB, Peres GL, Weber J, Zanelatto C, Koehnlein EA (2022). Effects of a Cereal Bar with a Combination of Phytoestrogens on the Climacteric Symptoms: A Placebo-Controlled, Randomized Trial. Journal of the American Nutrition Association.

[ref-16] Gencturk N, Bilgic FS, Kaban HU (2024). The effect of soy isoflavones given to women in the climacteric period on menopausal symptoms and quality of life: systematic review and meta-analysis of randomized controlled trials. Explore.

[ref-17] Gignac GE, Szodorai ET (2016). Effect size guidelines for individual differences researchers. Personality and Individual Differences.

[ref-18] Gomes MP, Deitcher SR (2004). Risk of venous thromboembolic disease associated with hormonal contraceptives and hormone replacement therapy: a clinical review. Archives of Internal Medicine.

[ref-19] Greene JG (1998). Constructing a standard climacteric scale. Maturitas.

[ref-20] Groeneveld FP, Bareman FP, Barentsen R, Dokter HJ, Drogendijk AC, Hoes AW (1993). The climacteric and well-being. Journal of Psychosomatic Obstetrics & Gynecology.

[ref-21] Higgins JPT, Cochrane Collaboration (2019). Cochrane handbook for systematic reviews of interventions.

[ref-22] Hilditch JR, Lewis J, Peter A, Van Maris B, Ross A, Franssen E, Guyatt GH, Norton PG, Dunn E (1996). A menopause-specific quality of life questionnaire: development and psychometric properties. Maturitas.

[ref-23] Huser S, Guth S, Joost HG, Soukup ST, Kohrle J, Kreienbrock L, Diel P, Lachenmeier DW, Eisenbrand G, Vollmer G, Nothlings U, Marko D, Mally A, Grune T, Lehmann L, Steinberg P, Kulling SE (2018). Effects of isoflavones on breast tissue and the thyroid hormone system in humans: a comprehensive safety evaluation. Archives of Toxicology.

[ref-24] Imhof M, Gocan A, Imhof M, Schmidt M (2018). Soy germ extract alleviates menopausal hot flushes: placebo-controlled double-blind trial. European Journal of Clinical Nutrition.

[ref-25] Johnson KA, Vemuri S, Alsahafi S, Castillo R, Cheriyath V (2016). Glycone-rich soy isoflavone extracts promote estrogen receptor positive breast cancer cell growth. Nutrition and Cancer.

[ref-26] Jou HJ, Wu SC, Chang FW, Ling PY, Chu KS, Wu WH (2008). Effect of intestinal production of equol on menopausal symptoms in women treated with soy isoflavones. International Journal of Gynaecology and Obstetrics.

[ref-27] Kang I, Rim CH, Yang HS, Choe JS, Kim JY, Lee M (2022). Effect of isoflavone supplementation on menopausal symptoms: a systematic review and meta-analysis of randomized controlled trials. Nutrition Research and Practice.

[ref-28] Khapre S, Deshmukh U, Jain S (2022). The impact of soy isoflavone supplementation on the menopausal symptoms in perimenopausal and postmenopausal women. Journal of Mid-life Health.

[ref-29] Khezri MR, Jafari R, Yousefi K, Zolbanin NM (2022). The PI3K/AKT signaling pathway in cancer: molecular mechanisms and possible therapeutic interventions. Experimental and Molecular Pathology.

[ref-30] Kupperman HS, Wetchler BB, Blatt MH (1959). Contemporary therapy of the menopausal syndrome. Journal of the American Medical Association.

[ref-31] Larson JS (1997). The MOS 36-item short form health survey. A conceptual analysis. Evaluation & the Health Professions.

[ref-32] Ma X, Li X, Ma L, Chen Y, He S (2021). Soy isoflavones alleviate polycystic ovary syndrome in rats by regulating NF-kappaB signaling pathway. Bioengineered.

[ref-33] Mainini G, Ercolano S, De Simone R, Iavarone I, Lizza R, Passaro M (2024). Dietary supplementation of myo-inositol, cocoa polyphenols, and soy isoflavones improves vasomotor symptoms and metabolic profile in menopausal women with metabolic syndrome: a retrospective clinical study. Medicina.

[ref-34] Moher D, Liberati A, Tetzlaff J, Altman DG, Group P (2009). Preferred reporting items for systematic reviews and meta-analyses: the PRISMA statement. Journal of Clinical Epidemiology.

[ref-35] Nahas EA, Nahas-Neto J, Orsatti FL, Carvalho EP, Oliveira ML, Dias R (2007). Efficacy and safety of a soy isoflavone extract in postmenopausal women: a randomized, double-blind, and placebo-controlled study. Maturitas.

[ref-36] Na Takuathung M, Teekachunhatean S, Chansakaow S, Klinjan P, Inpan R, Kongta N, Tipduangta P, Tipduangta P, Dukaew N, Sakuludomkan C, Koonrungsesomboon N (2024). The effects of SOY extract nutraceuticals on postmenopausal women’s health: A randomized, double-blind, placebo-controlled trial. Journal of Functional Foods.

[ref-37] Neshatbini Tehrani A, Hatami B, Helli B, Yari Z, Daftari G, Salehpour A, Hedayati M, Khalili E, Hosseini SA, Hekmatdoost A (2024). The effect of soy isoflavones on non-alcoholic fatty liver disease and the level of fibroblast growth factor-21 and fetuin A. Scientific Reports.

[ref-38] Nourozi M, Haghollahi F, Ramezanzadeh F, Hanachi P (2015). Effect of Soy Milk Consumption on Quality of Life in Iranian Postmenopausal Women. Journal of Family and Reproductive Health.

[ref-39] Ou Q, Zhang S, Fu C, Yu L, Xin P, Gu Z, Cao Z, Wu J, Wang Y (2021). More natural more better: triple natural anti-oxidant puerarin/ferulic acid/polydopamine incorporated hydrogel for wound healing. Journal of Nanobiotechnology.

[ref-40] Pop AL, Nasui BA, Bors RG, Penes ON, Prada AG, Clotea E, Crisan S, Cobelschi C, Mehedintu C, Carstoiu MM, Varlas VN (2023). The current strategy in hormonal and non-hormonal therapies in menopause—a comprehensive review. Life.

[ref-41] Poschner S, Maier-Salamon A, Zehl M, Wackerlig J, Dobusch D, Pachmann B, Sterlini KL, Jager W (2017). The impacts of genistein and daidzein on estrogen conjugations in human breast cancer cells: a targeted metabolomics approach. Frontiers in Pharmacology.

[ref-42] Ronis MJ (2016). Effects of soy containing diet and isoflavones on cytochrome P450 enzyme expression and activity. Drug Metabolism Reviews.

[ref-43] Secreto G, Chiechi LM, Amadori A, Miceli R, Venturelli E, Valerio T, Marubini E (2004). Soy isoflavones and melatonin for the relief of climacteric symptoms: a multicenter, double-blind, randomized study. Maturitas.

[ref-44] Sohn SI, Pandian S, Oh YJ, Kang HJ, Cho WS, Cho YS (2021). Metabolic engineering of isoflavones: an updated overview. Frontiers in Plant Science.

[ref-45] Stoer NC, Vangen S, Singh D, Fortner RT, Hofvind S, Ursin G, Botteri E (2024). Menopausal hormone therapy and breast cancer risk: a population-based cohort study of 1.3 million women in Norway. British Journal of Cancer.

[ref-46] Tao M, Shao H, Li C, Teng Y (2013). Correlation between the modified Kupperman Index and the menopause rating scale in chinese women. Patient Prefer Adherence.

[ref-47] Tranche S, Brotons C, Pascual de la Pisa B, Macias R, Hevia E, Marzo-Castillejo M (2016). Impact of a soy drink on climacteric symptoms: an open-label, crossover, randomized clinical trial. Gynecological Endocrinology.

[ref-48] Uifalean A, Schneider S, Ionescu C, Lalk M, Iuga CA (2015). Soy isoflavones and breast cancer cell lines: molecular mechanisms and future perspectives. Molecules.

[ref-49] Viscardi G, Back S, Ahmed A, Yang S, Mejia SB, Zurbau A, Khan TA, Selk A, Messina M, Kendall CW, Jenkins DJ, Sievenpiper JL, Chiavaroli L (2025). Effect of soy isoflavones on measures of estrogenicity: a systematic review and meta-analysis of randomized controlled trials. Advances in Nutrition.

[ref-50] Wang J, He Z, Raghavan V (2023). Soybean allergy: characteristics, mechanisms, detection and its reduction through novel food processing techniques. Critical Reviews in Food Science and Nutrition.

[ref-51] Wu PS, Wang CY, Chen PS, Hung JH, Yen JH, Wu MJ (2021). 8-hydroxydaidzein downregulates JAK/STAT, MMP, oxidative phosphorylation, and PI3K/AKT pathways in K562 cells. Biomedicines.

[ref-52] Yamashita S, Lin I, Oka C, Kumazoe M, Komatsu S, Murata M, Kamachi S, Tachibana H (2022). Soy isoflavone metabolite equol inhibits cancer cell proliferation in a PAP associated domain containing 5-dependent and an estrogen receptor-independent manner. Journal of Nutritional Biochemistry.

