# Supplementary material for: Effects of soy isoflavones on menopausal symptoms in perimenopausal women: a systematic review and meta-analysis
Source: PeerJ. 2025 Jul 23;13:e19715. doi: 10.7717/peerj.19715 (PMC12296567; doi:10.7717/peerj.19715)
Supplement: Supplemental Information 2 [file peerj-13-19715-s002.pdf]

Query	Source	Results	Date
(isoflavones[MH] OR flavones[MH] OR flavonoids[MH] OR genistein[MH] OR daidzein[MH] OR soy[TIAB] OR soya[TIAB])	PubMed	152497	10/20/24
(hot flash[TIAB] OR night sweat[TIAB] OR menopause[MH] OR menopaus[TIAB] OR post menopaus[TIAB] OR postmenopaus[TIAB] OR perimenopaus[TIAB] OR peri-menopaus[TIAB] OR peri menopaus[TIAB] OR climacteric[MH] OR climacteric[TIAB] OR Vaginal dryness[TIAB] OR mood swing[TIAB] OR unstable mood[TIAB] OR Postmenopausal Osteoporosis[MH] OR Postmenopausal Osteoporos[TIAB] OR Postmenopausal Bone Loss[TIAB] OR Perimenopausal Bone Loss[TIAB] OR Post Menopausal Osteoporos[TIAB] OR headache[MH] OR headache[TIAB] OR libido[MH] OR Decreased Libido[TIAB] OR loss of libido[TIAB] OR palpitations[TIAB] OR (depression[MH] AND menopause[MH]) OR (depression[TIAB] AND menopause[MH]) OR (anxiety[MH] AND menopause[MH]) OR (anxiety[TIAB] AND menopause[MH]) OR sleep problem[TIAB] OR sleep difficult[TIAB] OR difficult sleep[TIAB] OR problem sleep[TIAB])	PubMed	202880	10/20/24
(randomized controlled trial[PT] OR controlled clinical trial[PT] OR clinical trial[PT] OR clinical study[PT] OR randomized[TIAB] OR placebo[TIAB] OR randomly[TIAB] OR trial[TIAB] OR blind[TIAB] OR groups[TIAB] OR controlled clinical trials as topic[MH] OR randomized controlled trial[MH] OR placebos[MH] OR double-blind method[MH])	PubMed	4469914	10/20/24
(isoflavones[MH] OR flavones[MH] OR flavonoids[MH] OR genistein[MH] OR daidzein[MH] OR soy[TIAB] OR soya[TIAB]) AND (hot flash[TIAB] OR night sweat[TIAB] OR menopause[MH] OR menopaus[TIAB] OR post menopaus[TIAB] OR postmenopaus[TIAB] OR perimenopaus[TIAB] OR peri-menopaus[TIAB] OR peri menopaus[TIAB] OR climacteric[MH] OR climacteric[TIAB] OR Vaginal dryness[TIAB] OR mood swing[TIAB] OR unstable mood[TIAB] OR Postmenopausal Osteoporosis[MH] OR Postmenopausal Osteoporos[TIAB] OR Postmenopausal Bone Loss[TIAB] OR Perimenopausal Bone Loss[TIAB] OR Post Menopausal Osteoporos[TIAB] OR headache[MH] OR headache[TIAB] OR libido[MH] OR Decreased Libido[TIAB] OR loss of libido[TIAB] OR palpitations[TIAB] OR (depression[MH] AND menopause[MH]) OR (depression[TIAB] AND menopause[MH]) OR (anxiety[MH] AND menopause[MH]) OR (anxiety[TIAB] AND menopause[MH]) OR sleep problem[TIAB] OR sleep difficult[TIAB] OR difficult sleep[TIAB] OR problem sleep[TIAB]) AND (randomized controlled trial[PT] OR controlled clinical	PubMed	541	10/20/24

trial[PT] OR clinical trial[PT] OR clinical study[PT] OR randomized[TIAB] OR placebo[TIAB] OR randomly[TIAB] OR trial[TIAB] OR blind[TIAB] OR groups[TIAB] OR controlled clinical trials as topic[MH] OR randomized controlled trial[MH] OR placebos[MH] OR double-blind method[MH])			
(MeSH descriptor: [Isoflavones] explode all trees) or (isoflavones OR flavones OR flavonoids OR genistein OR daidzein OR soy OR soya):ti,ab,kw (Word variations have been searched)	Cochrane	5832	10/20/24
(MeSH descriptor: [Menopause] explode all trees) or (hot flash OR night sweat OR menopause OR menopaus OR post menopaus OR postmenopaus OR perimenopaus OR peri-menopaus OR peri menopaus OR climacteric OR Vaginal dryness OR mood swing OR unstable<br>mood OR Postmenopausal Osteoporosis OR Postmenopausal Osteoporos OR Postmenopausal Bone Loss OR Perimenopausal Bone Loss OR<br>Post Menopausal Osteoporos OR headache OR libido OR Decreased Libido OR loss of libido OR palpitations OR depression AND menopause OR anxiety AND menopause OR sleep problem OR sleep difficult OR difficult sleep OR problem sleep):ti,ab,kw<br>(Word variations have been searched)	Cochrane	81849	10/20/24
(MeSH descriptor: [Randomized Controlled Trial] explode all trees) or (controlled clinical trials as topic or clinical trial<br>or clinical study or placebos or double-blind method):ti,ab,kw (Word variations have been searched)	Cochrane	1264832	10/20/24
((MeSH descriptor: [Isoflavones] explode all trees) or (isoflavones OR flavones OR flavonoids OR genistein OR daidzein OR soy<br>OR soya):ti,ab,kw (Word variations have been searched)) AND<br>((MeSH descriptor: [Menopause] explode all trees) or (hot flash OR night sweat OR menopause OR menopaus OR post menopaus OR postmenopaus OR perimenopaus OR peri-menopaus OR peri menopaus OR climacteric OR Vaginal dryness OR mood swing OR unstable<br>mood OR Postmenopausal Osteoporosis OR Postmenopausal Osteoporos OR Postmenopausal Bone Loss OR Perimenopausal Bone Loss OR<br>Post Menopausal Osteoporos OR headache OR libido OR Decreased Libido OR loss of libido OR palpitations OR depression AND	Cochrane	913	10/20/24

menopause OR anxiety AND menopause OR sleep problem OR sleep difficult OR difficult sleep OR problem sleep):ti,ab,kw (Word variations have been searched)) AND (MeSH descriptor: [Randomized Controlled Trial] explode all trees) or (controlled clinical trials as topic or clinical trial or clinical study or placebos or double-blind method):ti,ab,kw (Word variations have been searched)			
TS=(isoflavones）OR AB=(flavones OR flavonoids OR genistein OR daidzein OR soy OR soya)	Web of Science	311163	10/20/24
TS=(Menopause) OR AB=(hot flash OR night sweat OR menopause OR menopaus OR post menopaus OR postmenopaus OR perimenopaus OR peri-menopaus OR peri menopaus OR climacteric OR Vaginal dryness OR mood swing OR unstable mood OR Postmenopausal Osteoporosis OR Postmenopausal Osteoporos OR Postmenopausal Bone Loss OR Perimenopausal Bone Loss OR Post Menopausal Osteoporos OR headache OR libido OR Decreased Libido OR loss of libido OR palpitations OR depression AND menopause OR anxiety AND menopause OR sleep problem OR sleep difficult OR difficult sleep OR problem sleep)	Web of Science	350394	10/20/24
TS=(randomized controlled trial) OR AB=(controlled clinical trial OR clinical trial OR clinical study OR randomized OR placebo OR randomly OR trial OR blind OR groups OR controlled clinical trials as topic OR randomized controlled trial OR placebos OR double-blind method)	Web of Science	11270894	10/20/24
(TS=(isoflavones）OR AB=(flavones OR flavonoids OR genistein OR daidzein OR soy OR soya)) AND (TS=(Menopause) OR AB=(hot flash OR night sweat OR menopause OR menopaus OR post menopaus OR postmenopaus OR perimenopaus OR peri-menopaus OR peri menopaus OR climacteric OR Vaginal dryness OR mood swing OR unstable mood OR Postmenopausal Osteoporosis OR Postmenopausal Osteoporos OR Postmenopausal Bone Loss OR Perimenopausal Bone Loss OR Post Menopausal Osteoporos OR headache OR libido OR Decreased Libido OR loss of libido OR palpitations OR depression AND menopause OR anxiety AND menopause OR sleep problem OR sleep difficult OR difficult sleep OR problem sleep)) AND (TS=(randomized controlled trial) OR AB=(controlled	Web of Science	459	10/20/24

clinical trial OR clinical trial OR clinical study OR randomized OR placebo OR randomly OR trial OR blind OR groups OR controlled clinical trials as topic OR randomized controlled trial OR placebos OR double-blind method))			
isoflavones'/exp OR isoflavones OR 'flavones'/exp OR flavones OR 'flavonoids'/exp OR flavonoids OR 'genistein'/exp OR genistein OR 'daidzein'/exp OR daidzein OR soy OR 'soya'/exp OR soya	Embase	329684	10/20/24
(('hot flash'/exp OR 'hot flash' OR (hot AND ('flash'/exp OR flash)) OR 'night sweat'/exp OR 'night sweat' OR (('night'/exp OR night) AND ('sweat'/exp OR sweat)) OR 'menopause'/exp OR menopause OR menopaus OR 'post menopaus' OR (post AND menopaus) OR postmenopaus OR perimenopaus OR 'peri menopaus' OR (peri AND menopaus) OR 'climacteric'/exp OR climacteric OR 'vaginal dryness'/exp OR 'vaginal dryness' OR (vaginal AND ('dryness'/exp OR dryness)) OR 'mood swing'/exp OR 'mood swing' OR (('mood'/exp OR mood) AND ('swing'/exp OR swing)) OR 'unstable mood' OR (unstable AND ('mood'/exp OR mood)) OR 'postmenopausal osteoporosis'/exp OR 'postmenopausal osteoporosis' OR (postmenopausal AND ('osteoporosis'/exp OR osteoporosis)) OR 'postmenopausal osteoporos' OR (postmenopausal AND osteoporos) OR 'postmenopausal bone loss'/exp OR 'postmenopausal bone loss' OR (postmenopausal AND ('bone'/exp OR bone) AND ('loss'/exp OR loss)) OR 'perimenopausal bone loss' OR (perimenopausal AND ('bone'/exp OR bone) AND ('loss'/exp OR loss)) OR 'post menopausal osteoporos' OR (post AND menopausal AND osteoporos) OR 'headache'/exp OR headache OR 'libido'/exp OR libido OR 'decreased libido'/exp OR 'decreased libido' OR (decreased AND ('libido'/exp OR libido)) OR 'loss of libido'/exp OR 'loss of libido' OR (('loss'/exp OR loss) AND of AND ('libido'/exp OR libido)) OR palpitations OR 'depression'/exp OR depression) AND ('menopause'/exp OR menopause) OR 'anxiety'/exp OR anxiety) AND ('menopause'/exp OR menopause) OR 'sleep problem'/exp OR 'sleep problem' OR (('sleep'/exp OR sleep) AND problem) OR 'sleep difficult' OR (('sleep'/exp OR sleep) AND difficult) OR 'difficult sleep' OR (difficult AND ('sleep'/exp OR sleep)) OR 'problem sleep' OR (problem AND ('sleep'/exp OR sleep))	Embase	465406	10/20/24

controlled clinical trial'/exp OR 'controlled clinical trial' OR (controlled AND ('clinical'/exp OR clinical) AND ('trial'/exp OR trial)) OR 'clinical trial'/exp OR 'clinical trial' OR (('clinical'/exp OR clinical) AND ('trial'/exp OR trial)) OR 'clinical study'/exp OR 'clinical study' OR (('clinical'/exp OR clinical) AND ('study'/exp OR study)) OR randomized OR 'placebo'/exp OR placebo OR randomly OR 'trial'/exp OR trial OR 'blind'/exp OR blind OR groups OR 'controlled clinical trials as topic'/exp OR 'controlled clinical trials as topic' OR (controlled AND ('clinical'/exp OR clinical) AND trials AND as AND topic) OR 'randomized controlled trial'/exp OR 'randomized controlled trial' OR (randomized AND controlled AND ('trial'/exp OR trial)) OR 'placebos'/exp OR placebos OR 'double-blind method'/exp OR 'double-blind method' OR ('double blind' AND ('method'/exp OR method))	Embase	18074068	10/20/24
('isoflavones'/exp OR isoflavones OR 'flavones'/exp OR flavones OR 'flavonoids'/exp OR flavonoids OR 'genistein'/exp OR genistein OR 'daidzein'/exp OR daidzein OR soy OR 'soya'/exp OR soya) AND ((('hot flash'/exp OR 'hot flash' OR (hot AND ('flash'/exp OR flash)) OR 'night sweat'/exp OR 'night sweat' OR (('night'/exp OR night) AND ('sweat'/exp OR sweat)) OR 'menopause'/exp OR menopause OR menopaus OR 'post menopaus' OR (post AND menopaus) OR postmenopaus OR perimenopaus OR 'peri menopaus' OR (peri AND menopaus) OR 'climacteric'/exp OR climacteric OR 'vaginal dryness'/exp OR 'vaginal dryness' OR (vaginal AND ('dryness'/exp OR dryness)) OR 'mood swing'/exp OR 'mood swing' OR (('mood'/exp OR mood) AND ('swing'/exp OR swing)) OR 'unstable mood' OR (unstable AND ('mood'/exp OR mood)) OR 'postmenopausal osteoporosis'/exp OR 'postmenopausal osteoporosis' OR (postmenopausal AND ('osteoporosis'/exp OR osteoporosis)) OR 'postmenopausal osteoporos' OR (postmenopausal AND osteoporos) OR 'postmenopausal bone loss'/exp OR 'postmenopausal bone loss' OR (postmenopausal AND ('bone'/exp OR bone) AND ('loss'/exp OR loss)) OR 'perimenopausal bone loss' OR (perimenopausal AND ('bone'/exp OR bone) AND ('loss'/exp OR loss)) OR 'post menopausal osteoporos' OR (post AND menopausal AND osteoporos) OR 'headache'/exp OR headache OR 'libido'/exp OR libido OR 'decreased libido'/exp OR 'decreased libido' OR (decreased AND ('libido'/exp OR libido)) OR 'loss of libido'/exp OR 'loss of libido' OR (('loss'/exp OR loss) AND of AND ('libido'/exp OR libido)) OR palpitations OR 'depression'/exp OR depression) AND ('menopause'/exp OR menopause) OR 'anxiety'/exp OR anxiety) AND ('menopause'/exp OR menopause) OR 'sleep problem'/exp OR 'sleep problem' OR (('sleep'/exp OR sleep) AND problem) OR 'sleep difficult' OR (('sleep'/exp OR sleep)	Embase	186	10/20/24

AND difficult) OR 'difficult sleep' OR (difficult AND ('sleep'/exp OR sleep)) OR 'problem sleep' OR (problem AND ('sleep'/exp OR sleep))) AND ('controlled clinical trial'/exp OR 'controlled clinical trial' OR (controlled AND ('clinical'/exp OR clinical) AND ('trial'/exp OR trial)) OR 'clinical trial'/exp OR 'clinical trial' OR (('clinical'/exp OR clinical) AND ('trial'/exp OR trial)) OR 'clinical study'/exp OR 'clinical study' OR (('clinical'/exp OR clinical) AND ('study'/exp OR study)) OR randomized OR 'placebo'/exp OR placebo OR randomly OR 'trial'/exp OR trial OR 'blind'/exp OR blind OR groups OR 'controlled clinical trials as topic'/exp OR 'controlled clinical trials as topic' OR (controlled AND ('clinical'/exp OR clinical) AND trials AND as AND topic) OR 'randomized controlled trial'/exp OR 'randomized controlled trial' OR (randomized AND controlled AND ('trial'/exp OR trial)) OR 'placebos'/exp OR placebos OR 'double-blind method'/exp OR 'double-blind method' OR ('double blind' AND ('method'/exp OR method)))			
