# Supplementary material for: Effects of soy isoflavones on menopausal symptoms in perimenopausal women: a systematic review and meta-analysis
Source: PeerJ. 2025 Jul 23;13:e19715. doi: 10.7717/peerj.19715 (PMC12296567; doi:10.7717/peerj.19715)
Supplement: Supplemental Information 3 [file peerj-13-19715-s003.docx]

| Author | Design | Country | N randomized subjects (Soy isoflavones,comparison) | Age (range)(Soy isoflavones,comparison) | Exposure | Daily dosage | Other compounds | Duration of active treatment | Outcome (measures) |
| --- | --- | --- | --- | --- | --- | --- | --- | --- | --- |
| Atteritano et al.2013 | Double-blind RCT,parallel | Italy | 389(198,191 placebo) | (49-67)(53.00±2.00,52.00±2.00) | Genistein | 54mg | Calcium carbonate (500 mg) Vitamin D (400 IU) | 2 years | Zung Self-Rating Depression Scale Scorea |
| Jou et al.2008 | Open label,parallel | China | 64(34,30 placebo ) | 53.8±3.8 EP group  53.6±3.8 non-EP group 54.3±2.8 placebo | Soy isoflavones | 135mg | None | 6 months | Modified Kupperman Indexb |
| Mansoreh et al.2015 | Double-blind RCT,parallel | Iran | 80(40,40 placebo) | (45-60)(52.13±3.05,51.39±2.89) | Soy isoflavones | 185.55mg | Calcium carbonate (500 mg) Vitamin D (200 IU) | 8 months | MENQOLc |
| Rilva et al.2009 | Double-blind RCT,parallel | Brazil | 84(42,42 placebo) | (45-60)(53.35±3.62) | Soy isoflavones | 120mg | None | 16 weeks | CES-Dd |
| Costa et al.2017 | Double-blind RCT,parallel | Brazil | 36(19,17 placebo) | (45-60)(56.0±1.3,52.7±1.3) | Soy isoflavones | 100mg | None | 10 weeks | Kupperman Indexe Menopause Rating Scalef Cervantes Scaleg |
| Frigo et.al.2021 | Double-blind RCT,parallel | Brazil | 48(24,24 placebo) | (40-65)(51±5.2,50.7±5.7) | Soy isoflavones | 40.5mg | Phytoestrogens derived from flaxseed(40.9mg) | 90 days | Kupperman index |
| Giorgio et.al.2005 | Double-blind RCT,parallel | Italy | 117(58,59 placebo) | (≥35)(52.0,52.0) | Soy isoflavones | 80mg | None | 3 months | Greene Climacteric Scaleh Greene Psychological Subscalei |
| Salvador et.al.2016 | Open label,parallel | [England](https://fanyi.so.com/?src=onebox# England) | 90(45,45 placebo) | (≥45)(51.8±3.1,51.5±3.5) | Soy isoflavones | 50mg | None | 12 weeks | Menopause Rating Scale |
| Malkanthi et.al.2010 | Double-blind RCT,parallel | Canada | 83(41,42 placebo) | (40-65)(53.39±5.05,53.50±4.44) | Genistein | 30mg | None | 12 weeks | Greene Climacteric Scale |
| Martin et.al.2018 | Double-blind RCT,parallel | Austria  Romania  Germany | 192(97,95 placebo) | (40-70)(54.3±6.4,53.6±5.3) | Soy isoflavones | 100mg | None | 12 weeks | Greene Climacteric Scale |
| Fontvieille et.al.2017 | Double-blind RCT,parallel | Canada | 31(15,16 placebo) | (50-70)(60.4±3.4,58.2±5.7) | 44 mg of daidzein 16 mg of glycitein 10 mg of genistein | | None | 12 months | SF-36j  Kupperman index PSS-10k |
| Mingkwan et.al.2024 | Double-blind RCT,parallel | Thailand | 100(50,50 placebo) | (48-65)(55.92±3.52,56.88±3.62) | Soy isoflavones | 209mg | Grape seed extract  Tomato extract (Lycopersicon esculentum Mill)  Vitamins Minerals | 12 weeks | Modified Kupperman Index MENQOL |
| aThis is a self-reporting scale consisting of 20 items to assess the presence and severity of depressive symptoms. bAn adaptation of the original Kupperman Index, used to assess the severity of menopausal symptoms. cThe Menopause-Specific Quality of Life questionnaire is a tool designed to measure the impact of menopause on a woman's quality of life. dThe Center for Epidemiologic Studies Depression Scale is a self-reporting questionnaire used to measure symptoms of depression in the general population. eA widely used scale to assess the severity of menopausal symptoms, which was one of the first tools developed for this purpose. fA validated instrument used to evaluate the severity of climacteric symptoms and the effectiveness of treatments for menopausal symptoms. gA 31-item questionnaire used to assess the health-related quality of life in women during the menopausal transition. hA self-assessment tool consisting of 21 items across five dimensions to evaluate menopausal symptoms, including anxiety, depression, somatic symptoms, vasomotor symptoms, and sexual function. iA part of the Greene Climacteric Scale that focuses on psychological symptoms such as anxiety and depression. jA multipurpose, short-form health survey with 36 questions about perceptions of health and well-being. ^k^The Perceived Stress Scale-10 is a self-report measure developed to assess the global level of perceived stress. | | | | | | | | | |
