# Supplementary material for: Effects of soy isoflavones on menopausal symptoms in perimenopausal women: a systematic review and meta-analysis
Source: PeerJ. 2025 Jul 23;13:e19715. doi: 10.7717/peerj.19715 (PMC12296567; doi:10.7717/peerj.19715)
Supplement: Supplemental Information 4 [file peerj-13-19715-s004.docx]

This article is intended for a diverse audience, including:

****Healthcare professionals****: Physicians, gynecologists, and nutritionists who specialize in women's health and menopause management. They can use this systematic review and meta-analysis to inform their clinical practice, especially when considering non-hormonal treatment options for menopausal symptoms.

****Researchers in the field of menopause and phytoestrogens****: Scientists and researchers studying the effects of soy isoflavones on menopausal symptoms. The study provides a comprehensive analysis of existing literature and can serve as a foundation for future research.

****Women experiencing perimenopause or menopause****: Women who are seeking alternative treatments for menopausal symptoms and are interested in understanding the potential benefits of soy isoflavones as a natural supplement.

****Public health policymakers****: Those who are involved in developing guidelines and recommendations for the management of menopause. This study can contribute to evidence-based decision-making regarding the use of soy isoflavones in public health initiatives.

****Pharmaceutical and supplement companies****: Companies that produce soy isoflavone supplements or related products. The findings can help them understand the efficacy of their products and guide future product development.

Overall, the article aims to provide a comprehensive and evidence-based assessment of soy isoflavones' role in managing menopausal symptoms, making it relevant to both clinical and research audiences as well as individuals directly affected by menopause.
